# The Putative C_2_H_2_ Transcription Factor MtfA Is a Novel Regulator of Secondary Metabolism and Morphogenesis in *Aspergillus nidulans*

**DOI:** 10.1371/journal.pone.0074122

**Published:** 2013-09-16

**Authors:** Vellaisamy Ramamoorthy, Sourabh Dhingra, Alexander Kincaid, Sourabha Shantappa, Xuehuan Feng, Ana M. Calvo

**Affiliations:** Department of Biological Sciences, Northern Illinois University, DeKalb, Illinois, United States of America; Universidade de Sao Paulo, Brazil

## Abstract

Secondary metabolism in the model fungus *Aspergillus nidulans* is controlled by the conserved global regulator VeA, which also governs morphological differentiation. Among the secondary metabolites regulated by VeA is the mycotoxin sterigmatocystin (ST). The presence of VeA is necessary for the biosynthesis of this carcinogenic compound. We identified a revertant mutant able to synthesize ST intermediates in the absence of VeA. The point mutation occurred at the coding region of a gene encoding a novel putative C_2_H_2_ zinc finger domain transcription factor that we denominated *mtfA*. The *A. nidulans mtfA* gene product localizes at nuclei independently of the illumination regime. Deletion of the *mtfA* gene restores mycotoxin biosynthesis in the absence of *veA,* but drastically reduced mycotoxin production when *mtfA* gene expression was altered, by deletion or overexpression, in *A. nidulans* strains with a *veA* wild-type allele. Our study revealed that *mtfA* regulates ST production by affecting the expression of the specific ST gene cluster activator *aflR*. Importantly, *mtfA* is also a regulator of other secondary metabolism gene clusters, such as genes responsible for the synthesis of terrequinone and penicillin. As in the case of ST, deletion or overexpression of *mtfA* was also detrimental for the expression of terrequinone genes. Deletion of *mtfA* also decreased the expression of the genes in the penicillin gene cluster, reducing penicillin production. However, in this case, over-expression of *mtfA* enhanced the transcription of penicillin genes, increasing penicillin production more than 5 fold with respect to the control. Importantly, in addition to its effect on secondary metabolism, *mtfA* also affects asexual and sexual development in *A. nidulans.* Deletion of *mtfA* results in a reduction of conidiation and sexual stage. We found *mtfA* putative orthologs conserved in other fungal species.

## Introduction

Fungal species produce numerous secondary metabolites [1], [2], [3], including compounds with detrimental effects, such as mycotoxins [4], capable of causing disease and death in humans and other animals [4], [5]. *Aspergillus nidulans*, a model filamentous fungus studied for more than fifty years, produces the mycotoxin sterigmatocystin (ST). This mycotoxin, ST, and the well-known carcinogenic compounds called aflatoxins (AF), produced by related species such as *A. flavus, A. parasiticus*, and *A. nomius* [6], are both synthesized through a conserved metabolic pathway [7], [8], [9] where ST is the penultimate precursor. The genes responsible for ST/AF production are clustered. Within these clusters, the regulatory gene *aflR* encodes a transcription factor that acts as a specific cluster activator [10], [11], [12].

The range of secondary metabolites produced by *A. nidulans* also includes bioactive compounds with demonstrated beneficial effects and applications for medical treatments, including antibiotics, such as the beta-lactam penicillin (PN) [13], [14], or anti-tumoral metabolites such as terrequinone [15], [16], with potential direct application in the medical field. In both cases the genes involved in the synthesis of these compounds are also found clustered [16], [17].

In fungi, secondary metabolism is often found to be governed by genetic mechanisms that also control asexual and sexual development [18]. One of these principal common regulatory links is the global regulatory gene *veA,* first described to be a developmental regulator in *A. nidulans* [19], [20]. In 2003 we described for the first time the connection between *veA* and the synthesis of diverse fungal secondary metabolites, including ST [21]. Absence of the *veA* gene in *A. nidulans* prevents *aflR* expression and concomitant ST biosynthesis. A similar effect was also observed in *Aspergillus flavus* and *Aspergillus parasiticus veA* deletion mutants, that lost the capacity to produce AFs [22], [23], [24]. Furthermore, *veA* also regulates the biosynthesis of other mycotoxins, for example cyclopiazonic acid and aflatrem in *Aspergillus flavus* [22]. *veA* is extensively conserved in Ascomycetes [25] and its global regulatory effect on mycotoxin biosynthesis was also observed in other fungal genera, for example, on the synthesis of trichothecenes in *F. graminerum* [26], and on fumonisins and fusarins in *Fusarium* spp, including *F. verticillioides* and *F. fujikuroi* [25], [27]; all these mycotoxins can cause severe impacts on the health of humans and other vertebrates [4]. Interestingly, VeA also regulates the production of other secondary metabolites with beneficial properties, for instance PN in *A. nidulans* and *P. chrysogenum* [21], [28] as well as cephalosporin C in *Acremonium chrysogenum* [29].

VeA has also been found to affect fungal infection of plants and animals. For example, a decrease in virulence was observed in deletion *veA* mutants of *A. flavus* when infecting plant tissue [24]. This effect was also observed in mycotoxigenic *Fusarium* species, such as *F. verticillioides* [25], *F. graminearum* [26] and *F. fujikuroi* [27]. In the case of animal infections, deletion of the *veA* homolog in *Histoplasma capsulatum* also leads to a reduction in virulence in a murine model [30], although in *Aspergillus fumigatus veA* is dispensable for virulence in the neutropenic mouse infection model [31].

Most of the studies to elucidate the *veA* regulatory mechanism of action have been carried out using the model fungus *A. nidulans*. It is known that the KapA α-importin transports the VeA protein to the nucleus, and that this transport is promoted by darkness [32], [33]. In the nucleus, VeA interacts with light-responsive proteins that also modulate mycotoxin production and fungal development, such as the red phytochrome-like protein FphA, which interacts with the blue sensing proteins LreA-LreB [34], [35]. VeA also sustains other nuclear protein interactions with VelB and LaeA [36], [37]. LaeA, a chromatin modifying protein, is also required for the synthesis of ST and other secondary metabolites [38], [39]. Absence of VelB, another protein of the *velvet* family [37], decreases and delays ST biosynthesis, indicating a positive role in ST biosynthesis [36].

To identify novel *veA*-dependent genetic elements involved in the regulation of ST biosynthesis in the model system *A. nidulans*, we performed a mutagenesis in a deletion *veA* strain to generate revertant mutants that regained the capacity to produce toxin [40]. Several revertant mutants (RM) were obtained. In the present study we characterized one of these selected revertants, RM7. This revertant mutant presented a point mutation in a gene that we denominated *mtfA* (*m*aster *t*ranscription *f*actor A) encoding a novel putative C_2_H_2_ zinc finger domain type transcription factor. We show that the *mtfA* effect on ST production is *veA*-dependent. Additionally, *mtfA* regulates the expression of other secondary metabolite gene clusters, such as those of terrequinone and PN. Furthermore, *mtfA* is also important for normal sexual and asexual development in *A. nidulans*.

## Materials and Methods

### Fungal Strains and Growth Conditions

Fungal strains used in this study are listed in Table 1. Media used include glucose minimal media (GMM) [41], YGT (0.5% yeast extract, 2% dextrose, trace elements prepared as described [41], and oat meal media (OMM) (1% oat meal). Supplements for auxotrophic markers were added as required [41]. Glucose was substituted with threonine (100 mM) in threonine minimal medium (TMM) for induction of *alcA* promoter. Solid medium was prepared by adding 10 g/liter agar. Strains were stored as 30% glycerol stocks at −80°C.

**Table 1:** Fungal strains used in the study.

Strain name	Pertinent genotype	Source
FGSC4	Wild type (*veA+*)	FGSC
RDAE206	*yA2, pabaA1, pyrG89; argB2,* Δ*stcE::argB,* Δ*veA::argB*	[40]
RDAEp206	*yA2*; Δ*stcE::argB*, Δ*veA::argB*	[40]
RAV1	*yA2, pabaA1, pyrG89; wA3; argB2,* Δ*stcE::argB; veA1*	[40]
RAV1p	*yA2; wA3*; Δ*stcE::argB; veA1*	[40]
RAV2	*yA2; wA3; argB2,* Δ*stcE::argB; pyroA4; veA1*	[40]
RM7	*yA2, pabaA1, pyrG89; argB2,* Δ*stcE::argB,* Δ*veA ::argB, mtfA*−	This study
RM7p	*yA2*, Δ*stcE::argB*, Δ*veA ::argB, mtfA−*	This study
RM7-R2	*yA2, pyrG89; wA3; argB2,* Δ*stcE::argB, mtfA*−, *veA1*	This study
RM7p-R2	*yA2; wA3*; Δ*stcE::argB, mtfA−, veA1*	This study
RM7-R2-com	*yA2, pyrG89; wA3; argB2*, Δ*stcE::argB, mtfA*−, *pRG3-AMA-NOT1-mtfA::pyr4; veA1*	This study
RJMP1.49	*pyrG89; argB2,* Δ*nkuA::argB; pyroA4; veA+*	[71]
TRV50.1	*argB2,* Δ*nkuA::argB; pyroA4; veA+*	This study
TRV50.2	*argB2,* Δ*nkuA::argB; veA+*	This study
TRVΔ*mtfA*	*pyrG89; argB2,* Δ*nkuA::argB;* Δ*mtfA::pyrG^A.fum^; pyroA4; veA+*	This study
TRVpΔ*mtfA*	Δ*nkuA::argB;* Δ*mtfA::pyrG^A.fum^; veA+*	This study
TRVΔ*mtfA*-com	*pyrG89; argB2,* Δ*nkuA::argB,* Δ*mtfA::pyrG^A.fum^; pyroA::mtfA; pyroA4; veA+*	This study
TRV60	*pyrG89; argB2,* Δ*nkuA::argB, alcA(p)::mtfA::pyr4; pyroA4; veA+*	This study
TDAEΔ*mtfA*	*pabaA1, pyrG89;* Δ*mftA::pyrG^A.fum^,* Δ*stcE::argB,* Δ*veA::argB*	This study
TDAEpΔ*mtfA*	*pyrG89;* Δ*mftA*::*pyrG^A.fum^,* Δ*stcE::argB,* Δ*veA::argB*	This study
RJW41.A	Δ*laeA; veA+*	[36]
RDIT2.3	*veA1*	[39]
RJW46.4	Δ*laeA; veA1*	[39]
RSD10.1	*pyrG89*; *wA3*; *argB2*, Δ*nkuA::argB*; Δ*mtfA*::*pyrG^A.fum^*; Δ*laeA::methG*; *veA1*	This study
RSD11.2	*pyrG89*; *wA3*; *argB2*, Δ*nkuA::argB*; Δ*mtfA*::*pyrG^A.fum^*; Δ*laeA::methG*; *veA+*	This study
TSD12.1	*pyrG89;* Δ*nkuA::argB; mtfA::gfp::pyrG^A.fum^; pyroA4*	This study

FGSC, Fungal Genetics Stock Center.

### Genetic Techniques

Meiotic recombination between *A. nidulans* strains was carried out as previously described [42]. Progeny from the cross between the RM7 mutant [40] and RAV2 (*yA2, wA3, argB2, ΔstcE::argB, pyroA4*) were analyzed for the presence or absence of *veA* by PCR. Colony morphology, as well as norsolorinic acid (NOR) production, were also studied. The progeny of this cross showed four phenotypic groups: 1. Δ*veA*, Δ*stcE*, X- (RM7 parental type); 2. Δ*stcE* (RAV2 parental type); 3. recombinant Δ*veA*, Δ*stcE* (RM7-R1) and recombinant Δ*stcE*, X- (RM7-R2). Dominance test was carried out by forming diploids with RM7-R2 and RAV1 strain.

### Identification of the Revertant Mutation in RM7

To find the mutation in RM7, *A. nidulans* genomic library pRG3-AMA1-NOT1 was utilized to transform the RM7-R2 (Δ*stcE*, X^−^) strain. Plasmid DNA was rescued from fungal transformants presenting wild-type phenotype. Both end regions of the DNA inserts in the isolated plasmids were sequenced and the complete insert sequences were found in the *A. nidulans* genome database (http://www.aspgd.org) by BLAST analysis. The exact location of the mutation in RM7 was identified by sequencing of the PCR product amplified from the same locus in RM7.

### Sequence Search and Alignment

The deduced protein sequence of MtfA (AN8741.2) was compared against databases from different fungal genera, using the BLAST (blastp) tool provided by National Center for Biotechnology Information (NCBI), (http://www.ncbi.nlm.nih.gov/). The gene entry with the highest percentage of identity and the lowest e-value for each of the species was selected (Table S1). Pairwise sequence alignment of the proteins was performed using the EMBOSS Needle tool (http://www.ebi.ac.uk/Tools/psa/emboss_needle/) from EMBL-EBI (European Molecular Biology Laboratory’s European Bioinformatics Institute). Percentage of similarities and percentage of identities were tabulated for each of the alignments (Table S1). Multiple sequence alignment was performed with MtfA (*A. nidulans*) and orthologs found across various fungal species using MAFFT version 6.0 (http://mafft.cbrc.jp/alignment/server/index.html), followed by shading using the BoxShade tool version 3.21. for presentation (http://www.ch.embnet.org/software/BOX_form.html).

### Phylogenetic Analysis

Phylogenetic analysis was performed for the following 20 species: A. oryzae, A. flavus, A. kawachii, A. niger, A. terreus, N. fischeri, A. fumigatus, A. clavatus, A. nidulans, P. chrysogenum, P. marneffei, A. capsulatus, U. reesii, C. immitis, F. oxysporum, M. oryzae, N. tetrasperma, N. crassa, C. globosum and B. fuckeliana. Orthologs of MtfA (A.nidulans) were identified in the above described genomes by searching against each other using the BLAST (blastp) tool from NCBI. Multiple sequence alignment was performed using MUSCLE v3.8.31 [43]. The alignment was used to build a Hidden Markov model (HMM), followed by realignment of sequences against the generated HMM, using the hmmbuild and hmmalign tools in HMMER v3.0b2 (http://hmmer.org/). A maximum likelihood phylogeny reconstruction method implemented in the software PhyML v3.0 [44], [45], whose workflow is available at iPlant collaborative™ (http://www.iplantcollaborative.org/) was used for tree construction with default settings. The resulting tree was viewed using FigTree v1.4.0 (http://tree.bio.ed.ac.uk/software/figtree/). Midpoint rooting [46] of the tree was chosen in order to minimize the large distances from the root to any leaf. The numbers on the branches indicate the approximate likelihood branch support values in percentages [47].

### Generation of the *mtfA* Deletion Strain

The entire *mtfA* coding region was replaced in RDAE206 and RJMP1.49 strains (Table 1). The DNA cassette used to delete *mftA* by gene replacement with the *pyrG* marker was obtained from FGSC (http://www.fgsc.net). Polyethylene glycol-mediated transformation of RDAE206 and RJMP1.49 protoplasts was carried out as described previously [48]. Transformants were selected on appropriate selection medium without uridine or uracil and confirmed by Southern blot analysis as previously described [49]. The deletion strains were designated as TDAEΔ*mtfA* and TRVΔ*mtfA* respectively.

A complementation strain was also obtained by transforming Δ*mtfA* (TRVΔ*mtfA*) strain with the *mtfA* wild-type allele. The complementation vector was generated as follows: A DNA fragment contained the entire *mtfA* coding region and 5′ and 3′ UTRs was first amplified with primers RM7com1 and RM7com2 (Table 2) from FGSC4 *A. nidulans* genomic DNA. Then the PCR product was digested with *Sac*II and *Kpn*I and cloned into pSM3 vector, containing the *pyroA* transformation marker, previously digested with the same enzymes, resulting in the plasmid pSM3-rm7com. This vector was transformed into Δ*mtfA* protoplasts and the transformants were selected on appropriate selection medium without pyridoxine. Complementation was confirmed by PCR and Southern blot analysis. The complemented strain was designated as Δ*mtfA-*com. Strains that were isogenic with respect to the auxotrophic markers were generated and used in this study.

**Table 2:** Primers used in this study.

Name	Sequence (5′→ 3′)
RM7-F1	TACGGCGATTCACTCACTTGGGC
RM7-R1	TAACTTACGCATGAGAAGCAGCCG
RM7com1	AAAAAACCGCGGGGATCTGCACTAGGAGATTG
RM7com2	AAAAAAGGTACCGACCGTGATACCTGATCTTC
RM7-OE1	AAAAAAGGTACCATGGATCTCGCCAACCTCATC
RM7-OE2	AAAAAATTAATTAATTACACCATCGCGACAGCCC
actin-F	ATGGAAGAGGAAGTTGCTGCTCTCGTTATCGACAATGGTTC
actin-R	CAATGGAGGGGAAGACGGCACGGG
aflR- F	GAGCCCCCAGCGATCAGC
aflR-R	CGGGGTTGCTCTCGTGCC
stcU-F	TTATCTAAAGGCCCCCCCATCAA
stcU-R	ATGTCCTCCTCCCCGATAATTACCGTC
nsdD-F	CATCTCACCAGCCACAATTACAGGCGGAACCATCAC
nsdD-R	TTGCGAGCCAGACACAGAGGTCATAACAGTGCTTGC
steA-F	TCCAGCAAATGGAACCGTGGAATCAGGTGCTC
steA-R	GAAGGGATGGGGCAAGAATGAGACTTCTGCGGGTAA
brlA-F	AGCTGCCTGGTGACGGTAGTTGTTGTTGGTGTTGC
brlA-R	CAGGAACGAATGCCTATGCCCGACTTTCTCTCTGGA
acvA_F	GACAAGGACAGACCGTGATGCAGGAGA
acvA_R	CCCGACGCAGCCTTAGCGAACAAGAC
aatA_F	CCATTGACTTCGCAACTGGCCTCATTCATGGCAAA
aatA_R	GCCTTCCGGCCCACATGATCGAAGAC
tdiAF	GCCCCAAGTCCATTGTCCTCGTTCAC
tdiAR	TCTGCGCCTGCTCGAGAGCAGCATC
tdiBF	CATGGACCCTACAGCACTCCTTCCT
tdiBR	GCGCTCTCAAAGTTCCGCT
mtfAgfpF_787	CCCCACCTCATCTCCAGCATC
mtfAgfpR_788	CACCATCGCGACAGCCCT
mtfA3′F_789	CCAATTGTGTTACTCCACCTCCTCG
mtfA3′R_790	TTGAGATCGCTTGCGCTCCTAG
mtfAlinkerF_791	AGGGCTGTCGCGATGGTGACCGGTCGCCTCAAACAATGCTCT
mtfAlinkerR_792	CGAGGAGGTGGAGTAACACAATTGGGTCTGAGAGGAGGCACTGATGCG
aflR06038	ATGGAGCCCCCAGCGATCAGCCAG
aflR06039	TTGGTGATGGTGCTGTCTTTGGCTGCTCAAC
mtfA13015	GCCCTCACCCTCATCGGCAATG
mtfA13016	GGTCGTGGTTCTGCTGGTAGGGTGT

### Generation of *mtfA* Over-expression Strain

To generate the *mtfA* over-expression strain, the entire *mtfA* coding region was first amplified using the RM7-OE1 and RM7-OE2 primers (Table 2). The PCR product was then digested with *Kpn*I and *Pac*I and ligated into pmacro plasmid, containing the *A. nidulans alcA* promoter, *trpC* terminator and *pyrG* marker, resulting in the plasmid pMacroMtfAOE. The pMacroMtfAOE vector was transformed into RJMP1.49 and transformants were selected on appropriate selection medium without uridine and uracil, and confirmed by PCR using RM7-OE1 and RM7-OE2 primers (Table 2) and Southern blot analysis (data not shown). The resulting selected transformant was denominated TRV60.

### Toxin Analysis

Culture plates containing 25 mL of solid GMM or OMM with appropriate supplements were top-agar inoculated with approximately 5×10^6^ spores/mL. The cultures were incubated in the dark. Three cores (16 mm diameter) from each replicate plate were collected and extracted with chloroform. Alternatively, strains were grown in GMM liquid shaken cultures (10^6^ spores/mL) and incubated at 37°C. Twenty-four h and 48 h old culture supernatants were analyzed for ST and mycelia were collected for RNA analysis. For analysis of mycotoxin production in over-expression *mtfA* and control cultures, GMM was inoculated with conidia (10^6^ conidia/mL) from the *mtfA* over-expression strain (TRV60) or its isogenic control (TRV50.1), and incubated for 16 h at 37°C and 250 rpm. At that time, equal amounts of mycelia were then spread onto inducing medium TMM. Culture supernatants were collected for toxin analysis and mycelia were collected for RNA analysis 24 and 48 hrs after shift. Culture supernatants were also extracted with chloroform.

The chloroform extracts were dried overnight and then resuspended in 200 µl of chloroform. Samples were fractionated by silica gel thin-layer chromatography (TLC) using benzene and glacial acetic acid [95∶5 (v/v)] as solvent system for ST analysis and chloroform:acetone:n-hexane (85∶15:20) for NOR analysis. Aluminum chloride (15% in ethanol) was sprayed on the plates, that were subsequently baked for 10 min at 80°C. Both ST and NOR bands present on TLC plates were visualized under UV light (375-nm).

### Penicillin Analysis

The PN bioassay analysis was carried out as previously described [50] with some modifications, using *Bacillus calidolactis C953* as testing organism. Briefly, strains were inoculated with approximately 10^6^ spores mL^−1^ in 20 mL of seed culture medium, and incubated at 26°C for 24 h at 250 rpm. Mycelia were collected with Miracloth (Calbiochem, USA) and transferred to PN-inducing medium containing lactose, 40 g/L; corn steep liquid (50%), 40 g/L; KH2PO4, 7 g/L; and phenoxyacetic acid, 0.5 g/L; pH was adjusted to 6.0 with 10 M KOH. Cyclopentanone (10 mM) was added to induce expression of the *alcA* promoter when the *mtfA* over-expression strain and its isogenic control were used. Twenty mL of PN-inducing medium was inoculated with 1 mL of mycelia suspension (containing equal amounts of mycelium), and mycelial samples were collected at 24 h and 48 h of incubation for RNA analysis. After 96 h, the culture supernatants were collected for PN analysis. The experiment was carried out with three replicates. Three hundred mL of Tryptone-Soy Agar was inoculated with 20 mL of *B. calidolactis C953* culture and plated on three 150-mm-diameter Petri dishes. Supernatant aliquots of each culture supernatant were then added to 7-mm-diameter wells. Bacteria were cultured at 55°C for 16 h and inhibition halos were visualized and measured. To confirm that the observed antibacterial activity was due to the presence of PN and not to the presence of other fungal compounds in the supernatant, controls containing commercial penicillinase from *Bacillus cereus* (Sigma, MO, USA) were also used. A standard curve using various concentrations of PN G (Sigma, MO, USA) was utilized to determine PN concentration in each sample.

### Study of MtfA Subcellular Localization

*Aspergillus nidulans* RJMP1.49 strain (Table 1) was transformed with *mtfA*::*gfp*::*pyrG^A.fum^* as described previously [48]. Primers used in the generation of the fusion PCR product utilized for transformation are listed in Table 2. Plasmid p1439 [32] was used as template for the PCR amplification of the intermediate fragment. Correct integration was confirmed by PCR and Southern blot analysis (data not shown). Conidia from the selected transformant (i.e. TSD12.1, Table 1) were inoculated as described previously [32]. Briefly, conidia were allowed to germinate on coverslip submerged in Watch minimal medium [51] in light or dark. After 16 h samples were washed in 1×PBS and stained with DAPI (10 ng/mL) in 50% glycerol and 0.1% Triton X-100. Samples were observed with a Nikon Eclipse E-600 equipped with Nomarski optics and fluorochromes for GFP and UV using a 100×objective. Micrographs were taken using Hamamatsu ORCA-ER high sensitivity monochrome CCD camera using Microsuite 5 imaging software. The exposure time for DIC, DAPI and GFP was 50 ms, 200 ms and 1 s respectively.

### Morphological Studies

Plates containing 25 mL of solid GMM with the appropriate supplements were top-agar inoculated with 5 mL of top agar containing 10^6^ spores/mL of *A. nidulans* strains TRV50.2 control, Δ*mtfA* or Δ*mtfA*-com (Table 1). The cultures were incubated in dark or in light at 37°C. Cores were removed from each culture and homogenized in water. Conidia and Hülle cells were counted using a hemacytometer. Identical cores were taken to visualize cleistothecia under a dissecting microscope. To improve visualization of fruiting bodies, the cores were sprayed with 70% ethanol to remove conidiophores.

For radial growth analysis, each strain was point inoculated and incubated under light or dark conditions at 37°C for 6 days, when colony diameter was measured. Experiments were performed with three replicates.

### Gene Expression Analysis

Total RNA was extracted from lyophilized mycelia using RNeasy Mini Kit (Qiagen) or Trizol (Invitrogen), following the manufacturer’s instructions. Gene expression levels were evaluated by Northern blots or quantitative reverse transcription-PCR (qRT-PCR) analysis. The templates used for making probes for Northern blots were obtained as follows: *ipnA*, a 1.1-kb *Hin*dIII-*Eco*RI fragment of pUCHH(458) [52]; *aflR*, *stcU*, *aatA*, *acvA*, *dtiA*, and *dtiB* probe templates were amplified by PCR from *A. nidulans* genomic DNA with primers indicated in Table 2.

For qRT-PCR, 2 µg of total RNA was treated with RQ1 RNase-Free DNase (Promega). cDNA was synthesized with Moloney murine leukaemia virus (MMLV) reverse transcriptase (Promega). qRT-PCR was performed with the Applied Biosystems 7000 Real-Time PCR System using SYBR green dye for fluorescence detection. The primer pairs used for qRT-PCR are listed in Table 1. The expression data for each gene was normalized to the *A. nidulans* actin gene expression and the relative expression levels were calculated using the 2^−ΔCT^ method.

## Results

### Locus AN8741.2, Mutated in RM7, Encodes a Putative C_2_H_2_ Type Transcription Factor

**Figure 1:**
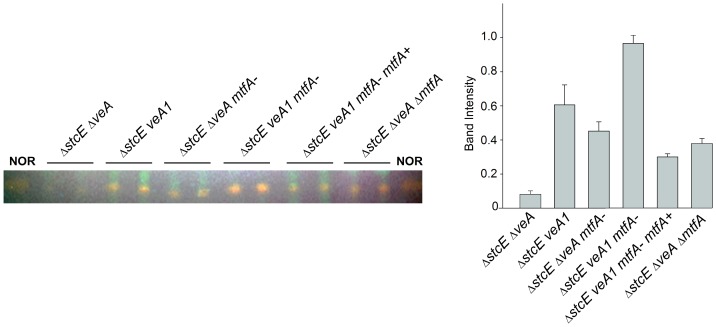
Revertant mutant 7 (RM7) produces NOR. TLC analysis of NOR production. Fungal strains were top-agar inoculated on OMM and incubated for six days. Then, mycelial cores were collected and NOR was extracted and analyzed as described in Materials and Methods section. Δ*stcE* Δ*veA* (RDAEp206), Δ*stcE veA1* (RAV1p), Δ*stcE* Δ*veA mtfA-* (RM7p), Δ*stcE veA1 mtfA*- (RM7p-R2), Δ*stcE veA1 mtfA*- *mtfA*+ (RM7p-R2-com), Δ*stcE* Δ*veA* Δ*mtfA* (TDAEpΔ*mtfA*). On the right, densitometry carried out with the Scion Image Beta 4.03 software.

**Figure 2:**
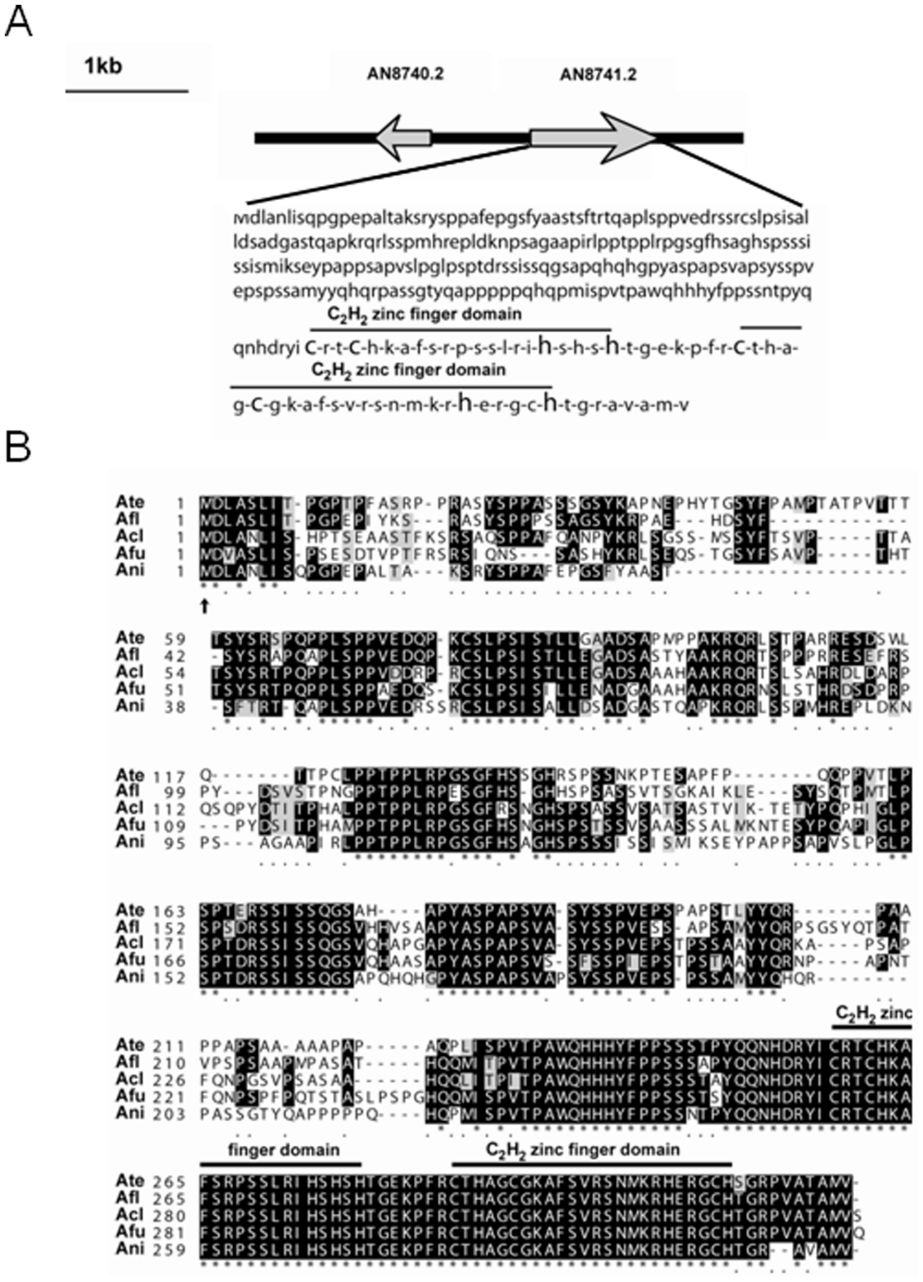
RM7 mutant presents a single gene mutation at locus AN8741.2. A) Diagram showing the genomic insert present in the complementation vector from library pRG3-AMA1-NOT1. The insert contains two ORFs corresponding to AN8741.2 and adjacent AN8740.2. The coding region at AN8741.2 locus encodes a putative C_2_H_2_ zinc finger domain transcription factor. The revertant mutation in RM7 occurred at AN8741.2, designated as *mtfA* gene. B) Amino acid alignment of *A. nidulans* MtfA (Ani) and putative orthologs in *A. terreus* (Ate), *A. flavus* (Afl), *A. clavatus* (Acl) and *A. fumigatus* (Afu). ClustalW (http://www.ebi.ac.uk/Tools/clustalw2/index.htm) land boxshade (http://www.ch.embnet.org/software/BOX_form.html) multiple sequence alignment software programs were utilized in this analysis. The mutation occurred at the codon corresponding to the first methionine. The two conserved zinc finger domains are indicated in A) and B).

In our previous study, we generated seven revertant mutants (RMs) capable of restoring normal levels in the production of the orange ST intermediate norsonolinic acid (NOR) in a Δ*stcE* strain lacking the *veA* gene (RDAE206) [40]. Classical genetics analysis revealed that these RMs belong to different linkage groups (data not shown). In the current study we identify the mutated gene in RM7 that restores toxin production in a deletion *veA* genetic background (Figure 1). The mutation in RM7 was recessive (data not shown) and the specific affected locus was found by complementation of RM7-R2 with an *A. nidulans* genomic library (pRG3-AMA1-NOT1, [53]).Several positive transformants showing wild-type phenotype were obtained. Sequencing of the rescued plasmids from these fungal transformants and comparison of these sequences with the *A. nidulans* genomic database (http://www.aspgd.org) by BLAST analysis indicated that they contained the same genomic insert including two ORFs, one of them encoding a putative C_2_H_2_ finger domain protein, and another encoding an unknown hypothetical protein (Figure 2). In order to determine where the mutation was located in RM7, the corresponding genomic DNA fragment was PCR-amplified. Sequencing of this PCR product revealed that the mutation occurred in a gene encoding the novel putative C_2_H_2_ transcription factor, that we designated *mtfA* (*m*aster *t*ranscription *f*actor A). The mutation was a G-T transversion at nucleotide +3 of the *mtfA* coding region, changing the start codon from ATG to ATT (Figure 2A).

### MtfA Orthologs are Present in Other Fungal Species

The deduced amino acid sequence of *A. nidulans* MtfA revealed significant identity with ortholog proteins from other *Aspergillus* spp., such as *A. clavatus* (64% identity), *A. terreus* (61%), *A. flavus* (61)%, or *A. fumigatus* (59%). Further analysis of other fungal genomic databases indicated that MtfA is also conserved in other fungal genera in Ascomycetes (Table S1, Figure S1 and S2). The C_2_H_2_ DNA binding domain is highly conserved among these putative orthologs. A MtfA ortholog was not found in the strict-yeast fungus *Saccharomyces cerevisiae*. Similarly, MtfA putative orthologs were not found in plants or animals. Orthologs from other fungal genera are listed in Table S1. An extensive alignment and phylogenetic tree is shown in Figure S1 and S2. MtfA orthologs were particularly conserved among *Aspergillus* spp. The MtfA tree topology was consistent with established fungal taxonomy. MtfA presents similarity to other *A. nidulans* C_2_H_2_ DNA binding domain proteins (Table S2), showing the highest similarity with FlbC (25.3% identity in the full protein comparison and 29% identity when comparing the DNA binding domains).

### *mtfA* Regulates Mycotoxin Biosynthesis

To confirm that NOR production in RM7 (Δ*veA*, X-) was indeed due to a loss-of-function mutation in *mtfA,* and to assess the effect of this mutation on ST production in a strain with a wild-type *veA* allele (*veA*+), we performed a complete deletion of *mtfA* in RDAE206 (Δ*veA*) and RJMP1.49 (*veA+*), obtaining TDAEΔ*mtfA* and TRVΔ*mtfA* strains, respectively (Figure S3). Deletion of *mtfA* in these strains was confirmed by Southern blot analysis, using the 5′ UTR as probe template P1 (Figure S3B). This probe revealed a 7.1 kb *Pst*I fragment in the wild-type control and a 6.3 kb *Pst*I fragment in the deletion mutants as expected. Also, hybridization with the transformation marker gene used for gene replacement, *AfpyrG* (specific probe template P2), revealed 6.3 kb and 2.2 kb *Pst*I fragments in *mtfA* deletion mutants, while these bands were absent in the wild-type control (Figure S3B), as predicted.

Similarly to RM7p (Δ*stcE*, Δ*veA, mtfA*-) (p, indicates prototrophy), the TDAEpΔ*mtfA* (Δ*stcE*, Δ*veA,* Δ*mftA*) strain shows an increase in NOR production with respect to RDAEp206 (Δ*stcE,* Δ*veA*), (Figure 1). The mutation in *mtfA* also allowed NOR production in a strain with a *veA1* allele, RM7-R2p (Δ*stcE, veA1, mtfA*−), a common *veA* mutant genetic background used in numerous *A. nidulans* research laboratories that still allows ST production. The levels of NOR production by RM7-R2p were similar to those detected in the isogenic control RAV1p (Δ*stcE, veA1*) (Figure 1).

**Figure 3:**
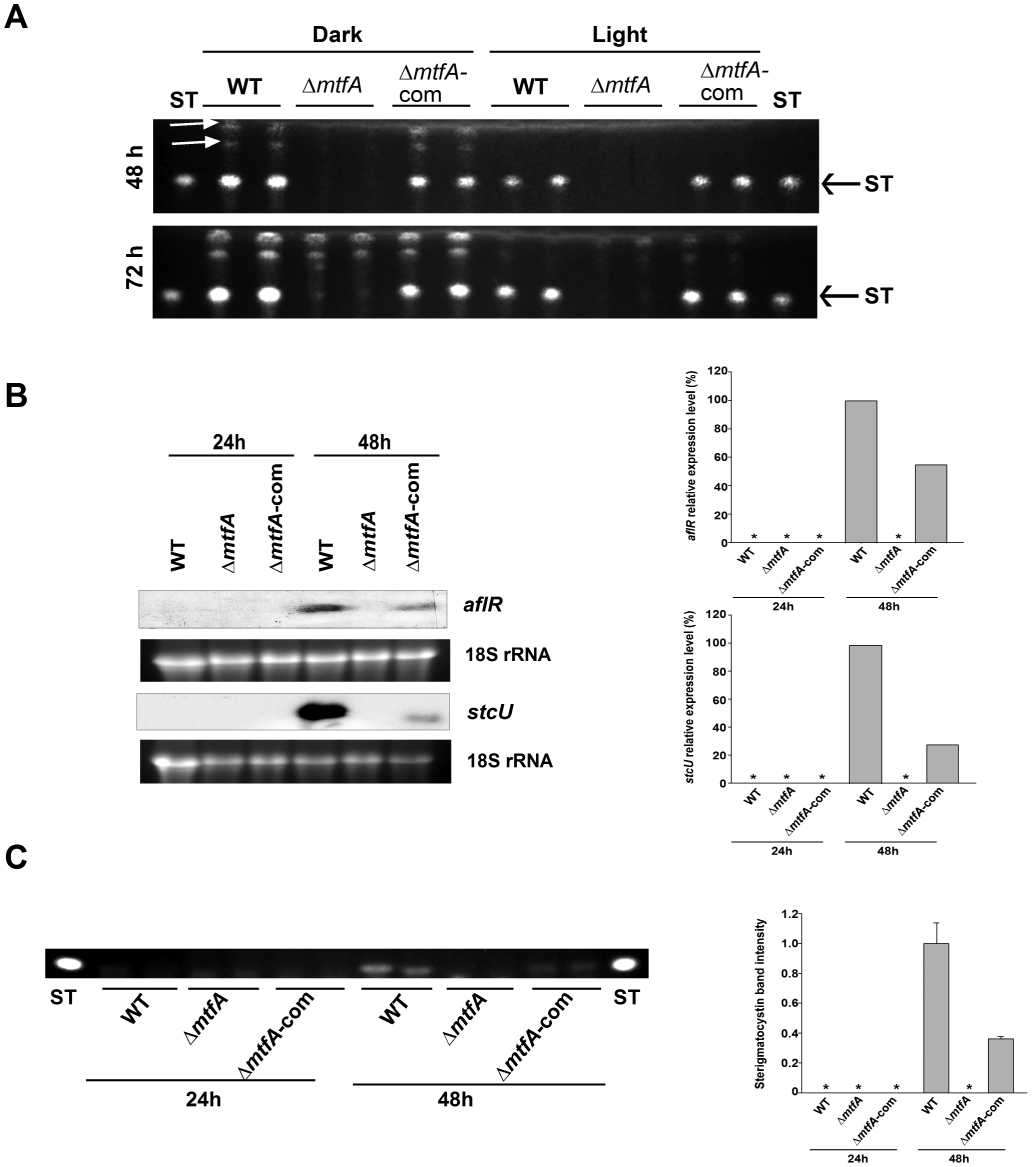
Effects of *mtfA* deletion on ST production in *A*. *nidulans* strains with a *veA+* allele. A) TLC analysis showing ST production in GMM cultures. Wild type (WT) *veA*+ control (TRV50.2), Δ*mtfA* (TRVpΔ*mtfA*) and Δ*mtfA*-com complementation strain (TRVΔ*mtfA*-com) were spread-inoculated with 5 mL of top agar containing 10^6^ conidia mL^−1^ and incubated at 37°C in the dark or in the light for 48 h and 72 h. ST was extracted and analyzed by TLC as described in the Materials and Methods section. White arrows indicate unknown compounds whose synthesis is also affected by the presence or absence of *mtfA.* B) Effect of the *mftA* deletion on *aflR* and *stcU* expression. Wild type (WT) *veA*+ control (TRV50.2), Δ*mtfA* (TRVpΔ*mftA*) and Δ*mtfA*-com complementation strain (TRVΔ*mtfA*-com) were inoculated in liquid GMM. Mycelia were collected 24 h and 48 h after inoculation. Cultures were grown in a shaker incubator at 37°C at 250 rpm. Expression of *aflR* and *stcU* was analyzed by Northern blot. 18S rRNA serves as loading control. Asterisk indicates not detected. C) TLC showing accumulation of ST in the cultures described in (B). Densitometries were carried out with the Scion Image Beta 4.03 software.

To elucidate the role of *mtfA* in mycotoxin biosynthesis in a strain with a *veA* wild-type genetic background (*veA*+) we analyzed ST production in the TRVΔp*mtfA* strain and compared it with that of the isogenic wild-type control strain and the complementation strain. Interestingly, our results indicated that TRVpΔ*mtfA* mutant did not produce ST after 48 h of incubation under both light and dark conditions in the *veA* wild-type background, whereas the wild type and complementation strain produced clearly detectable levels of ST (Figure 3A). At 72 h only very low levels of ST were detected in the TRVΔ*mtfA*p culture under these experimental conditions (Figure 3A). In addition, the TLC analysis indicated that deletion of *mtfA* also resulted in a delay in the synthesis of two additional unknown compounds in cultures growing in the dark (Figure 3A).

### *mtfA* Controls *aflR* Expression and Activation of the ST Gene Cluster

**Figure 4:**
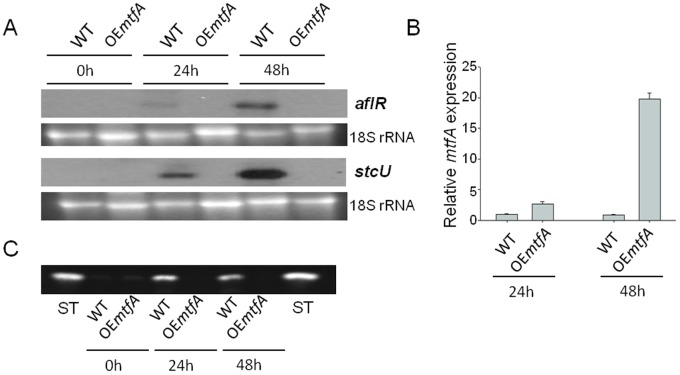
Over-expression of *mtfA* suppresses *aflR* and *stcU* expression and ST production. A) Northern blot analysis of *aflR* and *stcU* expression. Wild-type isogenic control (WT) *veA*+ (TRV50.1) and over-expression (OE) *mtfA* strain (TRV60) were inoculated in GMM liquid medium (10^6^ conidia mL^−1^) and grown for 16 hrs in a shaker incubator at 37°C and 250 rpm. Then, equal amounts of mycelium were transferred and spread onto TMM agar medium. The cultures were further grown for 48 h and 72 h. Mycelial samples were collected at 0 hr (shift time), and 24 and 48 hrs of incubation after shift onto TMM. 18S rRNA serves as loading control. B) qRT-PCR expression analysis of *mtfA* from mycelial samples collected after 24 h and 48 h of incubation after transfer onto TMM agar medium. C) TLC analysis of ST production from cultures described in (A-B).

Expression of the specific ST regulatory gene *aflR*, and expression of *stcU*, gene encoding a ketoreductase that is used as indicator for cluster activation [21], [54], were analyzed in liquid shaken cultures of wild type, deletion *mtfA* and complementation strain at 24 h and 48 h after spore inoculation. Neither *aflR* nor *stcU* were expressed in the *mtfA* deletion mutant, while transcripts for both genes accumulated at the 48 h time point analyzed (Figure 3B). The presence of these transcripts coincided with the presence of ST in the control cultures. Mycotoxin was not detected in the *mtfA* deletion cultures under the experimental conditions assayed (Figure 3C). Analysis of later time points also showed a notable reduction of ST production as well as a reduction in *aflR* expression in the Δ*mtfA* strain with respect to the controls (Figure S4), Over-expression of *mtfA* (*alcA(p)*::*mftA, veA+*) also prevented the transcription of *aflR* and *stcU* as well as ST production under conditions that allowed the control strains to activate the transcription of ST genes and mycotoxin production (Figure 4).

### Deletion of *mtfA* does not Recover Mycotoxin Biosynthesis in a Deletion *laeA* Genetic Background

Since VeA and LaeA proteins can interact in the nucleus and are, at least in part, functionally dependent, we examined whether loss of *mtfA* results in rescue of ST production in a Δ*laeA* strain. For this purpose, double Δ*mtfA*Δ*laeA* mutants were generated in *veA1* and *veA*+ genetic backgrounds by meiotic recombination from crosses between RJW34-1 (*pyrG89; wA3;* Δ*stcE::argB;* Δ*laeA*::*methG; trpC801; veA1*) and TRVΔ*mtfA* (Table 1). Our TLC analysis showed that deletion of *mtfA* did not recover ST biosynthesis in the strains with *laeA* deletion (Figure S5).

### *mtfA* Positively Regulates PN Biosynthesis by Controlling the Expression of the PN Gene Cluster

**Figure 5:**
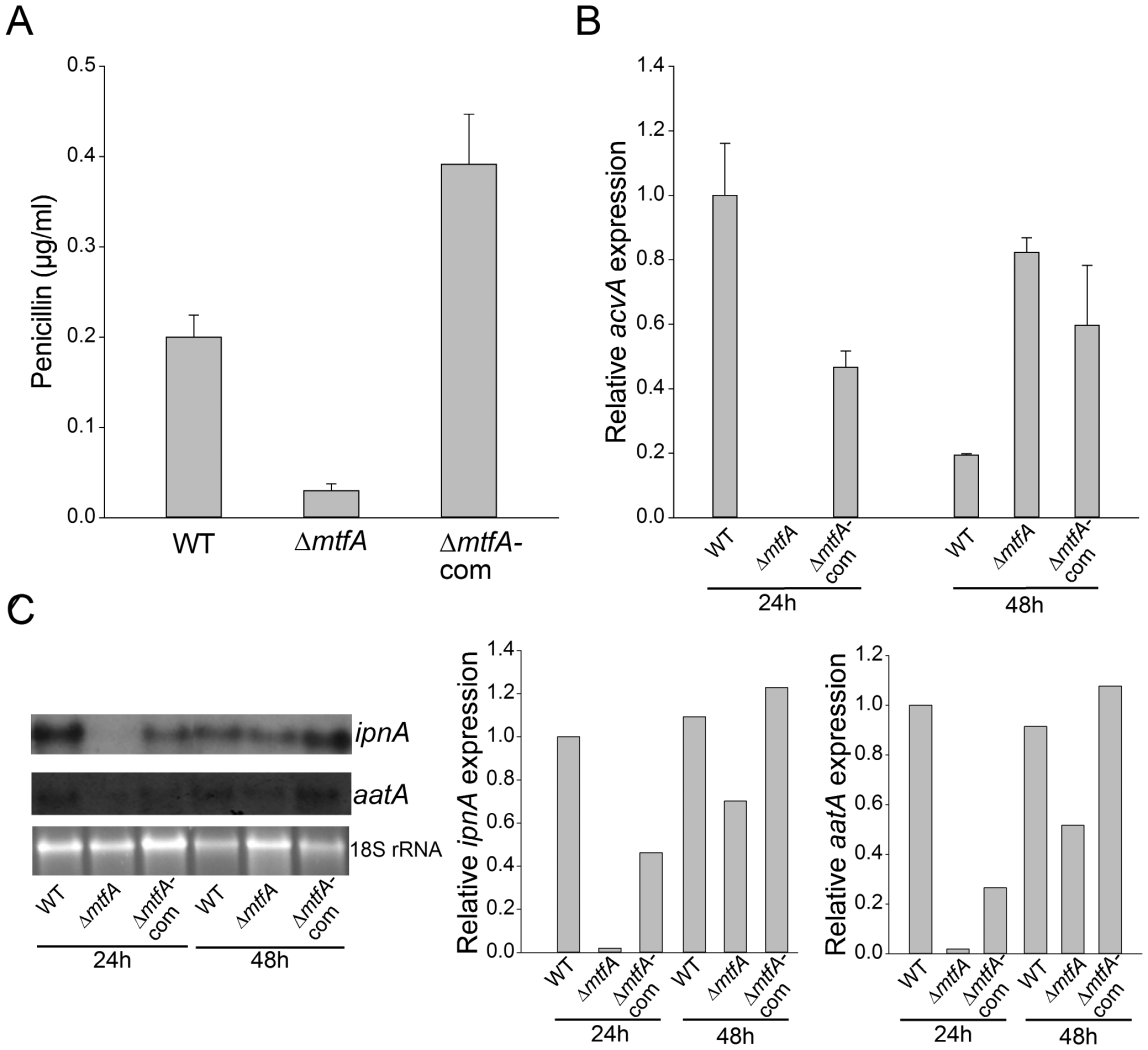
Deletion of *mtfA* results in a reduction of penicillin biosynthesis. A) Extracts from wild-type (WT) *veA*+ control (TRV50.2), Δ*mtfA* (TRVpΔ*mftA*) and Δ*mtfA*-com complementation strain (TRVΔ*mtfA*-com) were analyzed for penicillin content as described in Materials and Methods section. B) qRT-PCR expression analysis of *acvA* from mycelial samples collected after 24 h and 48 h of incubation in PN inducing medium. C) Northern blot analysis of *ipnA* and *aatA* from samples collected after 24 h and 48 h of incubation in PN inducing medium. Densitometries were carried out with the Scion Image Beta 4.03 software.

Results from our chemical analysis indicated that *mtfA* also affects the synthesis of other metabolites (Figure 5). Based on this finding, we also examined whether *mtfA* controls PN biosynthesis. We evaluated the production of this antibiotic in TRVpΔ*mtfA* and compared it with PN levels in the isogenic wild-type control and complementation strain. We used a strain of *B. calidolactis* as testing organism. Deletion of *mtfA* decreases penicillin production approximately 7-fold with respect to the wild type (Figure 5A), indicating that *mftA* is necessary for wild-type levels of penicillin biosynthesis. Our gene expression analysis revealed that *acvA, ipnA* and *aatA*, genes in the PN gene cluster [17], are down-regulated in the *mftA* deletion mutant (Figure 5B–C), particularly at the 24 h time point (24 h after mycelium is transferred to PN induction medium).

**Figure 6:**
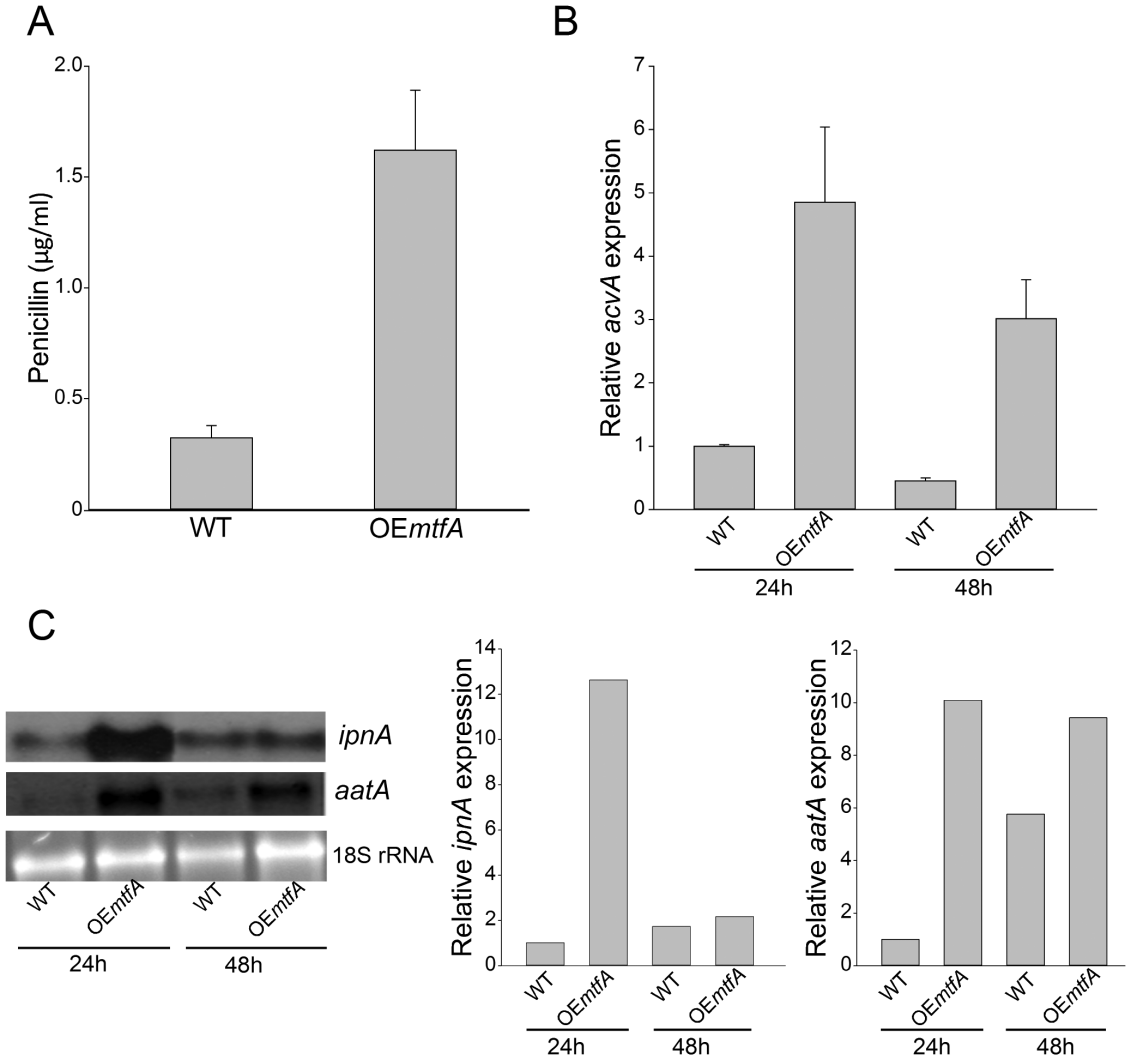
Over-expression of *mtfA* increases penicillin production. A) Extracts from wild-type (WT) *veA*+ control (TRV50.1), and over-expression (OE) *mtfA* strain (TRV60) were analyzed for penicillin content as described in Materials and Methods section. B) qRT-PCR expression analysis of *acvA* from mycelial samples collected after 24 h and 48 h of incubation in PN inducing medium. C) Northern blot analysis of *ipnA* and *aatA* from samples collected after 24 h and 48 h of incubation in PN inducing medium. Densitometries were carried out with the Scion Image Beta 4.03 software.

Over-expression of *mtfA* clearly increases production of PN (approximately 5-fold) with respect to the PN production levels obtained in the wild-type strain (Figure 6A). Expression of *acvA, ipnA* and *aatA*, was greater in the *mtfA* over-expression strain than in the control strain (Figure 6B–C). The experiment was repeated several times with similar results.

### *mtfA* Regulates the Expression of Terrequinone Genes

**Figure 7:**
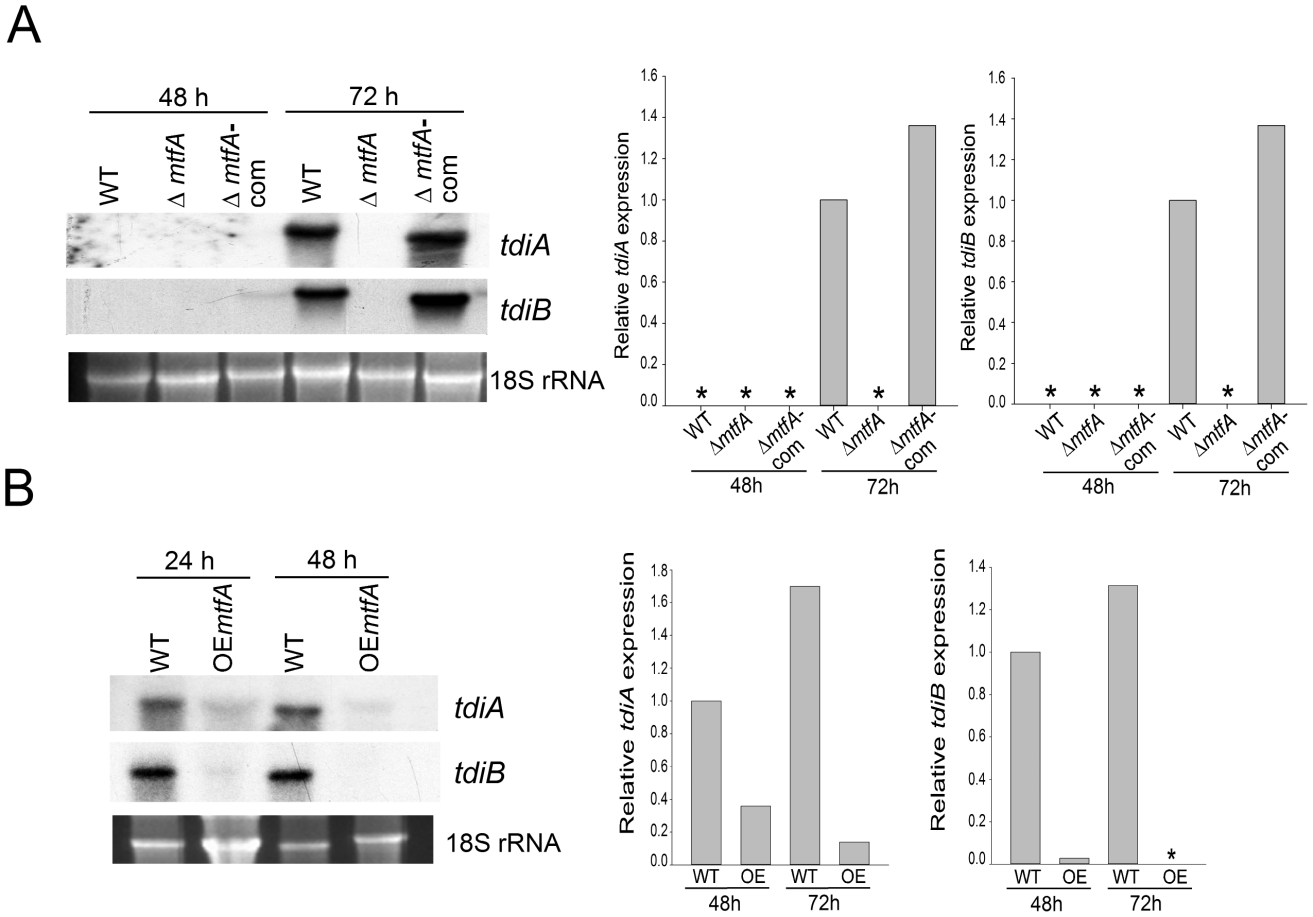
*mtfA* is necessary for normal expression of terrequinone genes. A) Wild type (WT) *veA*+ control (TRV50.2), Δ*mtfA* (TRVpΔ*mftA*) and Δ*mtfA*-com complementation strain (TRVΔ*mtfA*-com) were inoculated in liquid GMM. Mycelia were collected at 48 h and 72 h after inoculation for RNA extraction. Cultures were grown in a shaker incubator at 37°C at 250 rpm. Expression of *tdiA* and *tdiB* was analyzed by Northern blot. 18S rRNA serves as loading control. B) Isogenic wild type (WT) *veA*+ control (TRV50.1) and over-expression (OE) *mtfA* strain (TRV60) were inoculated in liquid GMM and grown for 16 h. After that, equal amounts of mycelium were transferred to TMM and further incubated for 24 h and 48 h. *tdiA* and *tdiB* expression was analyzed as in (B). Densitometries were carried out with the Scion Image Beta 4.03 software. Asterisk indicates not detected.

We also tested whether *mtfA* controls the expression of genes involved in terrequinone biosynthesis, a compound known for its anti-tumoral properties [15]. Specifically we examined the expression of *tdiA* and *tdiB* [16], [55]. At 24 h and 48 h of incubation, expression of *tdiA* and *tdiB* was detected in the wild-type control and complementation strains, while transcripts of these genes were absent in the *mtfA* deletion mutant (Figure 7A). Similarly to the case of ST production, over-expression of *mtfA* negatively affected the expression of *tdiA* and *tdiB* (Figure 7B)*;* Although transcripts were detected for both genes in the *mtfA* over-expression strain, *tdiA* expression levels were drastically reduced compared with the control at both 24 and 48 h after induction, and *tdiB* expression was only detected at 24 h in the over-expression *mtfA* at very low levels, while it was clearly detectable in the control strain at both time points analyzed (Figure 7B).

### MtfA Subcellular Localization

**Figure 8:**
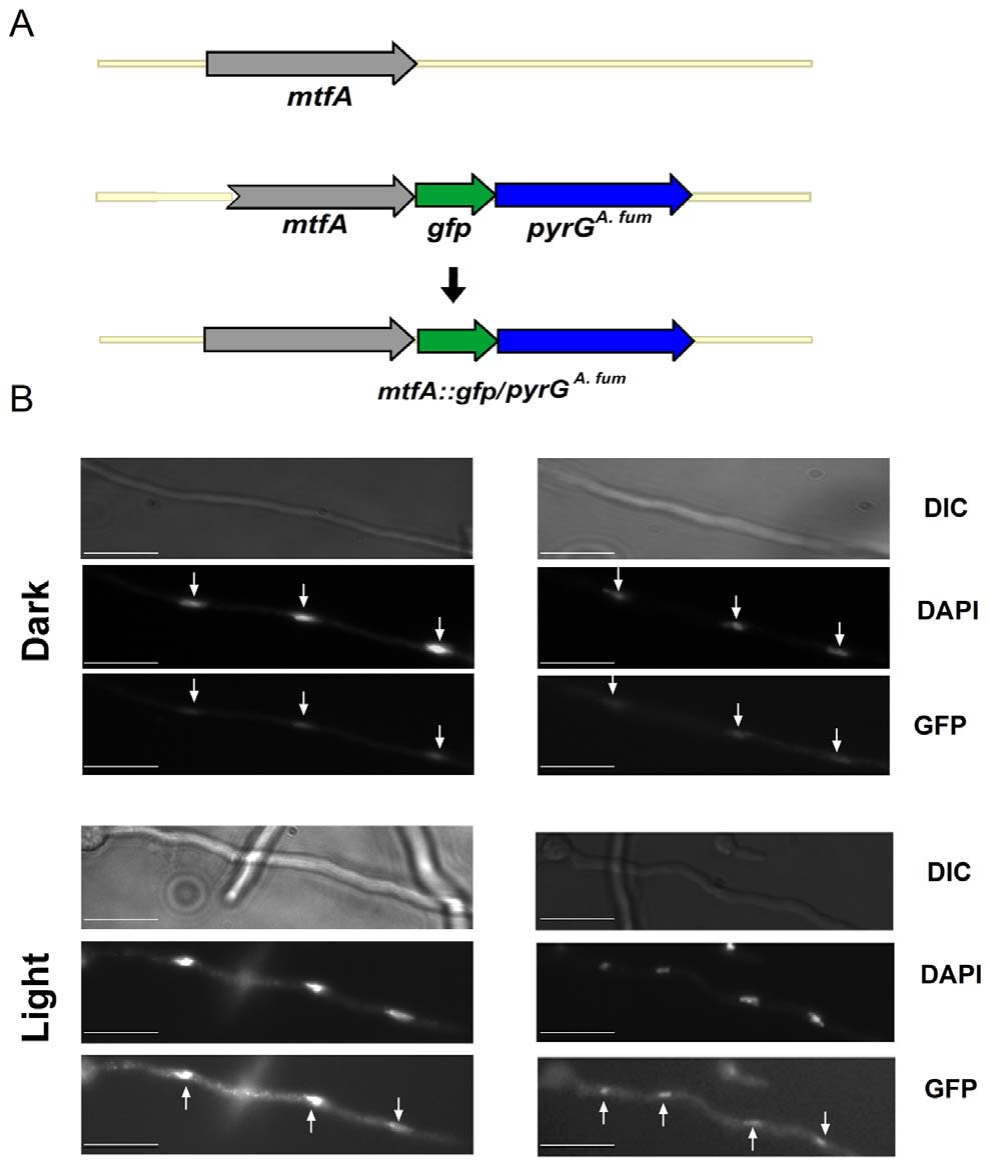
MftA localizes in nuclei. A) Diagram of the strategy utilized to fuse GFP to MtfA. The tagged construct was introduced at the *mtfA* locus by a double-over event. B) Micrographs showing the subcellular localization of the MtfA::GFP in *A. nidulans* growing in the light or in the dark. Scale bar represents 10 micrometers.

We further studied the function of the *A. nidulans mtfA* gene product by examining its subcellular localization in both light and dark conditions. Because the predicted MtfA has a C_2_H_2_ DNA binding domain we predicted that it could be found in nuclei. We generated a strain containing MtfA fused to GFP. Our observations using fluorescence microscopy indicated that indeed MftA localizes in nuclei, as revealed when compared with DAPI staining. Nuclear localization of MtfA was independent of the presence or absence of light (Figure 8).

### *mtfA* Regulates Asexual and Sexual Development in *A. nidulans*

**Figure 9:**
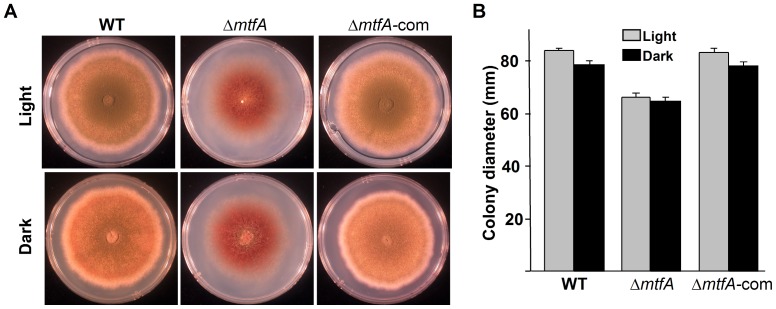
Deletion of *mtfA* affects fungal growth and colony pigmentation. A) Wild type (WT) *veA+* (TRV50.2), Δ*mtfA* (TRVpΔ*mtfA*) and Δ*mtfA*-com complementation (TRVΔ*mtfA*-com) were point inoculated on GMM plates and incubated at 37°C in either dark or light for 6 days. B) Fungal growth was measured as colony diameter. Values are means of four replicates. Standard error is shown.

**Figure 10:**
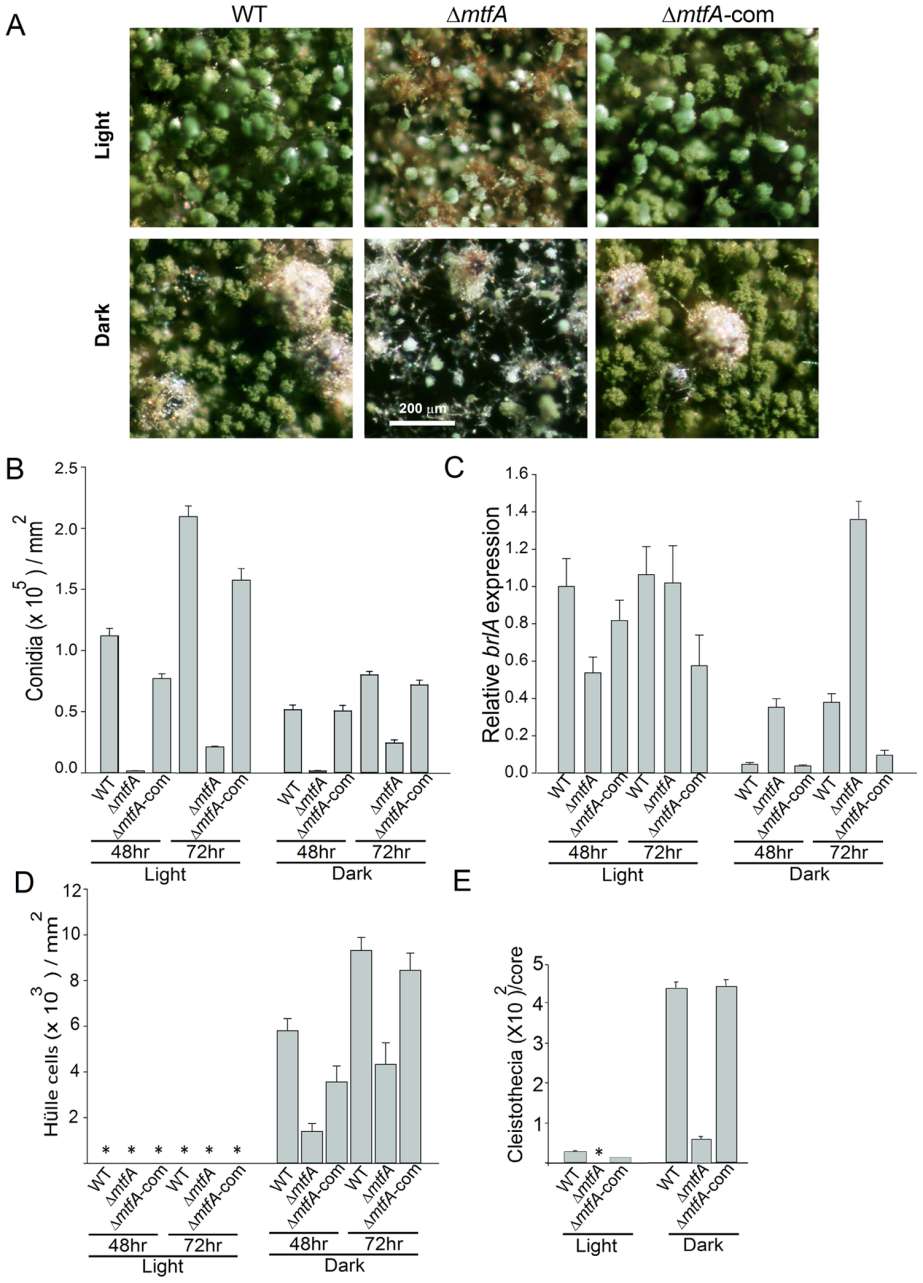
Deletion of *mtfA* mutant negatively affects conidiation and sexual development. **A)** Micrographs of point-inoculated cultures of wild type (WT) *veA*+ (TRV50.2), Δ*mtfA* (TRVpΔ*mtfA*) and Δ*mtfA*-com complementation (TRVΔ*mtfA*-com) strains grown in the light or in the dark for 6 days. Microscopy samples were collected 2 cm from the point of inoculation. Images were captured using upright Leica MZ75 stereomicroscope. B) Quantitative analysis of conidial production. Strains were top-agar inoculated (10^6^ conidia mL^−1^) and grown for 48 and 72 h on GMM. C) qRT-PCR quantification of *brlA* expression from the cultures described in (B). D) Quantitative analysis of Hülle cell production after 48 h and 72 h of incubation. E) Quantitative analysis of cleistothecial production after 10 days of incubation. Cleistothecia were counted after spraying the cultures with 70% ethanol to improve visualization. Core diameter was 16 mm. Asterisks in (D) and (E) indicate not detected. Values are means of three replicates Error bar indicates standard errors.

Deletion of *mtfA* results in slightly smaller colonies than the wild-type (Figure 9), indicating that *mtfA* positively influences fungal growth in both light and dark conditions. The *mtfA* deletion colonies presented a brownish pigmentation which is absent in the control strain. *mtfA* was expressed at similar levels under conditions promoting either asexual or sexual development, increasing transcript accumulation over time (Figure S6), Conidiophore formation and conidial production was drastically reduced in the *mtfA* deletion strains with respect to the wild type (Figure 10). This effect was observed in both light and dark cultures. The differences in conidiation levels were more pronounced in the light, a condition that promotes asexual development in *A. nidulans* [56]. In addition, the conidiophores produced by the Δ*mtfA* strain presented fewer metula and phialides than the control strains (Figure S7A). The reduction in conidiation observed in Δ*mtfA* coincided with alterations in the expression of *brlA* (Figure 10C), a key transcription factor in the initiation of conidiophore formation [57]. Reduction in *brlA* expression was observed after 48 h of incubation in the light, condition that promotes conidiophore formation. In the dark *brlA* levels in the wild type were low, as expected. However, expression of this gene in the *mtfA* mutant was abnormally high in the dark, a condition that represses conidiation [56]. The increase of *brlA* expression in Δ*mtfA* in the dark not only did not result in hyperconidiation, but the conidial production was as low as that observed in Δ*mtfA* growing in the light.

Sexual development is also influenced by *mtfA*. Absence of *mtfA* in *A. nidulans* results in a more than 2-fold reduction in Hülle cells, nursing cells participating in the formation of cleistothecia (fruiting bodies) (Figure 10D) [56]. Cleistothecial production was delayed and decreased in this mutant (Figures 10A, 10E and S7B-C). The cleistothecia present in Δ*mtfA* were of reduced size (Figure 10A). Expression of *nsdD* and *steA*, encoding transcription factors necessary for the activation of sexual development in *A. nidulans* [58], [59] did not significantly change in the absence of *mtfA* under the experimental conditions assayed (data not shown). Complementation of the deletion mutant with the *mtfA* wild-type allele restored wild-type morphogenesis.

## Discussion

This study revealed and characterized a new putative C_2_H_2_ transcription factor, MtfA. This protein, located in the cell nuclei, acts as master regulator in the production of several important secondary metabolites. In addition to this role, MtfA also affects asexual and sexual development in *A. nidulans*. MtfA presents two C_2_H_2_ zinc finger DNA-binding domains at the C-terminal region. In *A. nidulans* these C_2_H_2_ zinc finger domains have been found previously in other regulatory proteins, such as BrlA [57], SteA [60], PacC [52], SltA [61], CrzA [62], CreA [63] and FlbC [64]. Of the *A. nidulans* C_2_H_2_ zinc finger DNA-binding domain transcription factors examined, MtfA showed the highest similarity to FlbC with 25.3% identity. Our *in silico* analysis revealed that MtfA orthologs are present in many filamentous fungi, and they are not found in *S. cerevisiae* or in higher eukaryotes.

Our study indicated that *A. nidulans* MtfA controls the expression of *aflR*, a gene encoding another transcription factor specifically necessary for the activation of the ST gene cluster [10], [11], [12], and therefore, affecting the production of the ST toxin. We observed that either absence of *mtfA* or forced over-expression of *mtfA* results in a reduction of *aflR* transcription and decrease in ST biosynthesis, suggesting that only wild-type levels of *mtfA* gene product, in a balanced stoichiometry with other present factors, is conducive to normal ST levels. This delicate balance among regulatory factors has been previously observed with other regulators. For instance, in case of the global regulator VeA, where both deletion or over-expression of the gene encoding this protein were detrimental to the biosynthesis of the antibiotic PN [21], [65]. In addition, our study indicated that the *mtfA* role in regulating ST production is *veA*-dependent, which could, at least in part, explain the existence of these biological thresholds for proper function in the case of MtfA abundance in the cell.

VeA has been shown to be functionally associated with LaeA, a chromatin remodeling putative methyltransferase [39], [66], that forms part of the *velvet* complex in the nucleus [36], [37]; however, our study showed that deletion of *mtfA* did not suffice to rescue toxin production in strains where *laeA* is absent, in both *veA1* and *veA+* genetic backgrounds. This indicates that both *laeA* and *mtfA* are necessary for normal ST production in either *veA*+ or *veA1* background, Similar results were also observed in the case of the *A. nidulans rtfA* deletion mutant [40], suggesting that although VeA and LaeA are partially functionally connected, they also present differences in their regulatory output. It is possible that *mtfA* function could be associated with other components of the *velvet* complex. Future studies in our laboratory will provide further insight on *mtfA* mechanism of action and its possible connections with other known genetic regulatory networks.

Interestingly, *mtfA* showed a broad effect influencing the expression of other secondary metabolism gene clusters. Our results revealed that *mtfA* affects the expression of genes in the terrequinone gene cluster. In this case also both deletion or over-expression of *mtfA* lead to a reduction in the expression of *tdiA*, and *tdiB*, that encode asterriquinone synthetase and a protein with homology to fungal indole prenyltransferases, respectively, involved in the biosynthesis of terrequinone A, a known anti-tumoral bisindole alkaloid with applications in the medical field [16], [55].

In addition, our study showed that the master transcription factor, *mtfA*, also controls the expression of genes in the PN gene cluster and consequently the amounts of PN produced. While deletion of *mtfA* resulted in a reduction of PN production, differently from the case of ST and terrequinone A, *mtfA* over-expression enhances expression of PN genes, *acvA*, *ipnA* and *aatA*, and increases PN production with respect to the wild-type levels. This is relevant since PN and PN-derivatives have an important and well established commercial value, since these compounds are amongst the most important small molecules in clinical use for the last 70 years [67], [68], [69].

A relationship between the genetic regulation of fungal secondary metabolism and development has been previously observed [18], [35], [37], [40]. Our studies in *A. nidulans* indicated that *mtfA* not only regulates different aspects of secondary metabolism, but it also affects asexual and sexual development. The *mtfA* deletion strain presented a reduction in the number of conidiophores formed with respect to the wild type, leading to a notable decrease in conidial production in both light and dark cultures, and therefore reducing the potential of fungal dissemination. Analysis of the expression levels of *brlA*, an indispensable gene in the control of conidiophore formation [57], [70], indicated that *brlA* expression is dependent on *mtfA*. Furthermore, *A. nidulans* sexual development was also influenced by *mtfA*. The formation of Hülle cell was decreased in the *mtfA* deletion strain. This is relevant since Hülle cells are thick-walled cells that nourish cleistothecial primordia as they mature [19]. The decrease in Hülle cells observed at this early stage of sexual development could contribute to the delay and reduction in the number of mature cleistothecia formed in the *mtfA* deletion strain.

In conclusion, we found a novel master transcription factor, MtfA, that controls the activation of several secondary metabolism gene clusters and regulates asexual and sexual morphological development in *A. nidulans*. Our study indicated that MtfA is conserved in numerous filamentous fungi, particularly among Ascomycetes, many of them species of importance in industrial application, in agriculture or in the medical field. *mtfA* homologs were not found in plant or animal genomes, suggesting that *mtfA* or its gene product could have great potential to be used as a genetic target to reduce the detrimental effects of fungi, for instance production of mycotoxin, while enhancing those traits that are beneficial, such as increase in the production of antibiotics and other medical drugs of fungal origin.

## Supporting Information

Figure S1 **Alignment of MtfA-like proteins in filamentous fungi.** *Aspergillus nidulans* (*A.nidulans*), *Aspergillus oryzae* (*A.oryzae*), *Aspergillus niger* (*A.niger*), *Aspergillus kawachii* (*A.kawachii*), *Neosartorya fischeri* (*N.fischeri*), *Penicillium chrysogenum* (*P.chrysogenum*), *Coccidioides immitis* (*C.immitis*), *Ajellomyces capsulatus* (*A.capsulatus*), *Uncinocarpus reesii* (*U.reesii*), *Penicillium marneffei* (*P.marneffei*), *Botryotinia fuckeliana* (*B.fuckeliana*), *Neurospora tetrasperma* (*N.tetrasperma*), *Neurospora.crassa* (*N.crassa*), *Magnaporthe oryzae* (*M.oryzae*), *Chaetomium globosum* (*C.globosum*) and *Fusarium oxysporum* (*F.oxysporum*). Accession ID’s and source of these sequences are as mentioned in. MAFFT version 6.0 (http://mafft.cbrc.jp/alignment/server/index.html) and BoxShade version 3.2.1 (http://www.ch.embnet.org/software/BOX_form.html) were utilized for alignment and presentation.
(RTF)

Figure S2 **Maximum-Likelihood (ML) phylogenetic tree inferred from ortholog sequences of MtfA (** ***A.nidulans*** **) across genomes from several fungal species.** Protein alignment was done with MUSCLE; aLRT (approximate Likelihood Ratio Test) branch support values were calculated with PhyML v3.0 and the tree was plotted using FigTree v1.4.0. Only alRT branch support values >80% are indicated. The protein sequences used are as follows: *Aspergillus oryzae* (*A.oryzae*), *Aspergillus flavus* (*A.flavus*), *Aspergillus kawachii* (*A.kawachii*), *Aspergillus niger* (*A.niger*), *Aspergillus terreus* (*A.terreus*), *Neosartorya fischeri* (*N.fischeri*), *Aspergillus fumigatus* (*A.fumigatus*), *Aspergillus clavatus* (*A.clavatus*), *Aspergillus nidulans* (*A.nidulans*), *Penicillium chrysogenum* (*P.chrysogenum*), *Penicillium marneffei* (*P.marneffei*), *Ajellomyces capsulatus* (*A.capsulatus*), *Uncinocarpus reesii* (*U.reesii*), *Coccidioides immitis* (*C.immitis*), *Fusarium oxysporum* (*F.oxysporum*), *Magnaporthe oryzae* (*M.oryzae*), *Neurospora tetrasperma* (*N.tetrasperma*), *Neurospora crassa* (*N.crassa*), *Chaetomium globosum* (*C.globosum*) and *Botryotinia fuckeliana* (*B.fuckeliana*). NCBI (National center for Biotechnology Information) accession numbers for all sequences utilized in these analyses are shown in Table S1 in the supplemental material.
(TIF)

Figure S3 **Targeted** ***mtfA*** **deletion.** A) Diagram showing *Pst*I sites (P) in the wild-type *mtfA* locus, and the same locus after gene replacement of *mtfA* by the *A. fumigatus pyrG* gene (*AfpyrG*), used as selection marker for fungal transformation. Recombination events between the the flanking regions are indicated with crosses (X). Primers used for the construction of the deletion cassette are indicated by small arrows as described by FGSC. Fragments used as probe templates for Southern blot analyses are also shown. B) Southern blot analyses. The Δ*mtfA* deletion construct was transformed in RDAE206 and RJMP 1.49 strains (Table 1). *Pst*I digested genomic DNA of FGSC4 wild type (WT) and transformants, TDAEΔ*mtfA* (Δ*veA*, Δ*mtfA*) and TRVΔ*mtfA* (*veA+* Δ*mtfA*), was hybridized with probe P1, containing 5′ flanking sequence of *mtfA,* and probe P2, containing *AfpyrG* coding fragment. TDAEΔ*mtfA* transformants #1, 2 and 4 present the correct band pattern. TRVΔ*mtfA* transformants #1, 2 and 3 present the correct band pattern.
(TIF)

Figure S4 **Effects of** ***mtfA*** **deletion on ST production and** ***aflR*** **expression at late time points.** A) TLC analysis showing ST production in GMM cultures. Wild type (WT) *veA*+ control (TRV50.2), Δ*mtfA* (TRVpΔ*mtfA*) and Δ*mtfA*-com complementation strain (TRVΔ*mtfA*-com) were spread-inoculated with 5 mL of top agar containing 10^6^ conidia mL^−1^ and incubated at 37°C in the dark or in the light for 96 h and 120 h. ST was extracted and analyzed by TLC. B) Effect of the *mftA* deletion on *aflR* expression. Wild type (WT) *veA*+ control (TRV50.2), Δ*mtfA* (TRVpΔ*mftA*) and Δ*mtfA*-com complementation strain (TRVΔ*mtfA*-com) were inoculated in liquid GMM. Mycelia were collected 72 h and 96 h after inoculation. Cultures were grown in a shaker incubator at 37°C at 250 rpm. Expression of *aflR* was analyzed by qRT-PCR. A TLC showing accumulation of ST in these cultures and corresponding densitometry is also shown.
(TIF)

Figure S5 **Deletion of** ***mtfA*** **does not rescue mycotoxin production in Δ** ***laeA*** **strains.** TLC analysis of ST produced by the wild type (WT) *veA*+ control (TRV50.2), Δ*laeA veA+* (RJW41.A), Δ*mtfA veA+* (TRVpΔ*mtfA*) and Δ*mtfA* Δ*laeA veA+* strains (RSD11.2), *veA1* (RDIT2.3), Δ*mtfA veA1* (RJW46.4), Δ*mtfA* Δ*laeA veA1* (RSD10.1) grown on GMM at 37°C for 5 days.
(TIF)

Figure S6 **Expression of** ***mtfA*** **in the wild-type strain.** qRT-PCR analysis showing *mtfA* expression in the wild-type strain (TRV50.2) at the times indicated under conditions promoting asexual (light) or sexual development (dark). The strains were top-agar inoculated on GMM and incubated at 37°C.
(TIF)

Figure S7 **Micrographs of asexual and sexual structures.** A) Conidiophores forming in wild type (WT) *veA*+ (TRV50.2), Δ*mtfA* (TRVpΔ*mtfA*) and Δ*mtfA*-com complementation (TRVΔ*mtfA*-com) strains in top agar-inoculated solid GMM cultures incubated for 5 days in the light at 37°C. Bar represent 20 micrometers. CP, conidiophores. B) Micrographs showing the presence of cleistothecia (CL) in wild type (WT) *veA*+ (TRV50.2), and Δ*mtfA*-com complementation (TRVΔ*mtfA*-com) cultures growing in the dark for 5 days. Magnification 50×. C) Micrographs showing details of sexual structures. Bar represents 15 micrometers. CL, portion of an open cleistothecium; AS, ascospores; HC, Hülle cells.
(TIF)

Table S1 **Amino acid sequence comparison of** ***Aspergillus nidulans*** **MtfA in with putative orthologs in other fungal species.** The comparisons were done using the BLASTp tool provided by NCBI (National Center for Biotechnology Information) and EMBOSS Needle - Pairwise Sequence Alignment tool provided by EMBL-EBI (European Bioinformatics Institute).
(DOCX)

Table S2 **Comparison of MtfA with other** ***A. nidulans*** **C_2_H_2_ transcription factors.**
(DOC)
