# Supplementary material for: The Putative C2H2 Transcription Factor MtfA Is a Novel Regulator of Secondary Metabolism and Morphogenesis in Aspergillus nidulans
Source: PLoS One. 2013 Sep 16;8(9):e74122. doi: 10.1371/journal.pone.0074122 (PMC3774644; doi:10.1371/journal.pone.0074122)
Supplement: Figure S1 — Alignment of MtfA-like proteins in filamentous fungi. Aspergillus nidulans (A.nidulans), Aspergillus oryzae (A.oryzae), Aspergillus niger (A.niger), Aspergillus kawachii (A.kawachii), Neosartorya fischeri (N.fischeri), Penicillium chrysogenum (P.chrysogenum), Coccidioides immitis (C.immitis), Ajellomyces capsulatus (A.capsulatus), Uncinocarpus reesii (U.reesii), Penicillium marneffei (P.marneffei), Botryotinia fuckeliana (B.fuckeliana), Neurospora tetrasperma (N.tetrasperma), Neurospora.crassa (N.crassa), Magnaporthe oryzae (M.oryzae), Chaetomium globosum (C.globosum) and Fusarium oxysporum (F.oxysporum). Accession ID’s and source of these sequences are as mentioned in. MAFFT version 6.0 (http://mafft.cbrc.jp/alignment/server/index.html) and BoxShade version 3.2.1 (http://www.ch.embnet.org/software/BOX_form.html) were utilized for alignment and presentation. (RTF) [file pone.0074122.s001.rtf]

A.nidulans       1 MDLANLISQPGPE--PALTAKSRYSPPAFEPGSFYAAS---------------TSFTRT-
A.oryzae         1 MDLASLIT-PGPE--PIYKSRASYSPPPSSAGSYKRPAEH--DSYF--------SYSRAP
A.niger          1 MDLASLISHPGPD--PIMKSRASYSPPMT---SYKRSIEHTSDSYFPSVP---ISYTRSP
A.kawachii       1 MDLASLISHPGPD--PIMKSRASYSPPMT---SYKRSIEQTSDSYFPSVP---ISYTRSP
N.fischeri       1 MDVASLISPSESDTVPTFRSRSIQNSSAS---HYKRLSEQYTGSYFSAAPTHTTSYSRTP
P.chrysogenum    1 MDLSNLLSHSAAV-------KPIYTPVES------------------------SYYKRS-
C.immitis        1 MNVSSLITCDQPH--QLRAPASSYSEHR--------RSPSIPKPLQTESSSCASPYSRFE
A.capsulatus     1 MNLSHLVTSYHSP--PSTYPHSGTSQKRQ--SLQSESSLSVSNGYYDRNASN-LAYARSP
U.reesii         1 MNVSSLISCDQTA--PFHGSATSYFEHHQ-----RIRSPSIPKRSHEENSSSASPYPPFA
P.marneffei      1 MDNV--------------------------------------------------------
B.fuckeliana     1 M-ASSLVSNPYTV--HPMAQHSSY------------------------------TYVNAP
N.tetrasperma    1 M-APTTLT--------PQYPAQPY------------------------------GFA---
N.crassa         1 M-APTTLT--------PQYPAQPY------------------------------GFA---
M.oryzae         1 M-AATMIQQPYPI----HQQQSQY------------------------------SYMVQP
C.globosum       1 M-ANTMVTHYAHV---PQHSLQ-Y------------------------------GYM---
F.oxysporum      1 M-----------------------------------------------------------

A.nidulans      43 QAPLSPPVEDRSSRCSLPSISALLDSADGAST-------QAPKRQRLSSPMHREP-----
A.oryzae        48 QAPLSPPVEDQ-PKCSLPSISTLLEGADSAST-------YAAKRQRTSPPPRRES-----
A.niger         53 QPPLSPPVEDQSPKCSLPSISTLLEGADGAAM-------HAAKRTRMTPPLQRDL-----
A.kawachii      53 QPPLSPPVEDHSPKCSLPSISTLLEGADGAAM-------HAAKRTRMTPPLQRDL-----
N.fischeri      58 QPPLSPPAEDQ-PKCSLPSISILLENADGAAA-------HAAKRQRTSLSTHRDS-----
P.chrysogenum   29 -PPLSPPAEE--PKVSLPSISSLFEGADGAQHAATSLTLNLPERQRLSP-----------
C.immitis       51 RLPLSPPEEDGKTQFSLPSISSLLRGVDGVSDA------HVAKRQRTNPPPSIDL-----
A.capsulatus    56 QPPLSPPVEEQ-SRFSLPSISSLLQGADQLSPV------HIAKKHRPNPLSTGEV-----
U.reesii        54 TLPLSPPEDDGKTTFSLPSISSLLQSVDAASDT------HVAKRQRANPPPSIDL-----
P.marneffei      5 ---------------------------------------PASKRAR--------------
B.fuckeliana    28 QPPPSPPVDET-SKCSLPSISSLLGLADGSSPT------EQAQQQSSPQQAAF-------
N.tetrasperma   19 -PPPSPPLDDS-NKCSLPSISNLLVMADQGSPT------SETSPQSQQ------------
N.crassa        19 -PPPSPPLDDS-NKCSLPSISNLLVMADQGSPT------SETSPQSQQ------------
M.oryzae        26 QGPPSPPMDD--NKCSLPSISNLLGLADQGSPT------SETSAQFREEQKQQQA-----
C.globosum      23 -PPPSPPMDEA-AKCSLPSISNLLGLADQGSPT------SETSPQSQQQQQAQQQQQQQC
F.oxysporum      2 ---------EE-QKCSLPSISNLLGLADAGSPT------SESSPTSRQHSPRFEV-----

A.nidulans      91 --------------------------------LDKNPS------AGAAPIRLPPTPPLRP
A.oryzae        95 --------------------------------EFRSPY------DSVSTPNGPPTPPLRP
A.niger        101 --------------------------------DSRQQSQAYDLKANGPQIALPPTPPLRP
A.kawachii     101 --------------------------------DSRQQSQAYDLKANGPQIALPPTPPLRP
N.fischeri     105 ----------------------------------GPPY------DSITPHAMPPTPPLRP
P.chrysogenum   75 --------------------------------SLGDRH------VRVQSYELPPTPPLRP
C.immitis      100 --------------------------GMERR---TIDQ------TLKQRPALPLTPPLRP
A.capsulatus   104 ------------------------DLKSQGH-GATQKP------IHRPRMILPPTPPMRP
U.reesii       103 --------------------------ALERR-GACADQ------AIRQRPALPLTPPLRP
P.marneffei     12 -----------------------------------HDS------GDYSRGFLPPTPPMRP
B.fuckeliana    74 ---------------------KEDYRPESGH-QYGPSS------SMSSRGALPPTPPMQS
N.tetrasperma   59 -----------------LHFSKPDNRPNSSQ---FGNP------A-SIRANLPPSPPMSS
N.crassa        59 -----------------LHFSKPDNRPNSSQ---FGNP------A-SIRANLPPSPPMSS
M.oryzae        73 ---------------------AQQSRPNSSH---YSNA------VQSVRQGIPPTPPMTS
C.globosum      75 MSSSWWDMGHLDTDSTPAQGSKPETRPNSSH---YTNP------V-TIRTGLPPSPPMSS
F.oxysporum     41 ------------------------PPPSHGHSRAGSEW------AKSSHRGLPPTPPMST

A.nidulans     113 GSGFHSAGHSP-SSSISSISMI-----KSEYPAPPS------------APVSLPGLPSPT
A.oryzae       117 ESGFHSGHHSPSASSVTSGKAI----KLESYSQTPM------------------TLPSPS
A.niger        129 GSSFHSAGHSP-ASSISAASDAAAPKRSDSYPQVPM------------------ALPSPS
A.kawachii     129 GSSFHSAGHSP-ASSISAASDAAAPKRSDSYPQVPM------------------ALPSPS
N.fischeri     125 GSGFHSNGHSPSASSVSATSSSAVMKNTETYSQAPI------------------GLPSPT
P.chrysogenum   97 GSGHAHRRASP-VESLSHKEAH----------------------------------QHHL
C.immitis      125 ESGMNSTSQSPSTSSPPRSAIS-----LPSLVRSYPSPVSE-----VPEGRRMSQISRHS
A.capsulatus   133 GSGLDGRNHSPAGSSPSSAHSPISVANLTSSSSADPSYQHR-----MPQG----PLPPQS
U.reesii       130 ESGMGGVNHSPSASSPPRTAIS-----LPSLIGSYPSPVSE-----APEGRRMSQISRHS
P.marneffei     31 CSGFTEG-SSPASLPSGRSHSA-----SISSAVSHPSHQQRTSLPSISASLQNTPI-HPS
B.fuckeliana   106 DGGFDGR-QSPSQASTSSYSVV-SAP-NYYFNPSQVSAINN-----MEPHAQRQPVQTVT
N.tetrasperma   92 EASFEGY-RSPSSKPASQSQGS----SNYYYETTPPLSQ-------HEADSRQM--ATAA
N.crassa        92 EASFEGY-RSPSSKPASQSQGS----SNYYYETTPPLSQ-------HEADSRQM--ATAT
M.oryzae       103 ETSFDGY-NSPSNKSVSQLPAT-----GYYFEATPPPGH-------MEMEPRPH--MTSV
C.globosum     125 DASFEGF-NSPSTRSVSQVPNG----SNYFFETTPPL-Q-------MEADARQMTAAAAV
F.oxysporum     71 DASFEGY-SSPTRKPSNQAYPG-SAPRTYYYETTPP----------LEADAQRQASVTAI

A.nidulans     155 DRSSISSQGSAPQHQH----GPYASPAPSVAPSYSSPVEPSPS------SAMYY-QHQRP
A.oryzae       155 DRSSISSQGSVHHVSA----APYASPAPSVA-SYSSPVESSAP------SAMYY---QRP
A.niger        170 DRSSISSQGSVQGVSS----ASYASPAPSVS-SYSSPIEPSAS------SAMFY---QRT
A.kawachii     170 DRSSISSQGSVQGVSS----ASYASPAPSVS-SYSSPIEPSAS------SAMFY---QRT
N.fischeri     167 DRSSISSQGSVQHAAG----APYASPAPSVS-SFSSPVEPSTPS-----TAAYY---QRN
P.chrysogenum  122 HRSSISSNSSVHIPRN---TVPYASPVPSVS-SYTSPVDAPQ-------QPMYY---PRP
C.immitis      175 RGASTSQTSQLSGPET-----RYPSPPNVNSPTFAAPVEPAPK------PTEYY---PAS
A.capsulatus   184 TRSSVSQNSPVSLPEK---HYAPSSNLPTSSTPFASPVEPLAN------STEYY---HRP
U.reesii       180 SRTSISQSSQHPGPEA-----RYPSPPTLSSPSFAAPIEPPPK------PEYYS---SGA
P.marneffei     84 ERLSISSLAS-HDSSR----LSHAIPSP--SSTTASITTTATP------STSYYSTSEEK
B.fuckeliana   158 RRVSMPVSSMQYGHSPFNGSYTMSPGAQSLSSYYPSPIQTQSP----QVSSLYY---QRP
N.tetrasperma  138 PRAPVQSSTFQTQYPS----SAGYSSQSGMNPYYP-PMQPTPPPQQ-QMSGLYY---QRP
N.crassa       138 PRAPVQSSTFQTQYPS----SAGYSSQSGMNPYYP-PMQPTPPPQQ-QMSGLYY---QRP
M.oryzae       148 SRVPVQAPFAQSAYSA----PYGMAPSNPMAAYYP-TMQPTPPPQQPQISSLYY---QRP
C.globosum     172 PRVSVQASAYQPQYAP----GPAYMSQPAMTSYYP-PMQSAAPPQT-QMSGLYY---QRP
F.oxysporum    119 PRATPPATAPYPQQAH----PTVYANPAPVGAYYP-AAQVPPAVQP-QEMNPYY---QRP

A.nidulans     204 ASSGTYQ--AP--------------PP---PPQHQPMISPVT--PAWQHHHYFPPSSNTP
A.oryzae       201 --SGSYQ--TPAT---VPSPSAAPMPA---SATHQQMITPVT--PAWQHHHYFPPSSSAP
A.niger        216 ----------------APSTSAAPLPT---PAAPQQIISPVN--PAWQHHHYFPPSSTTP
A.kawachii     216 ----------------APSTSAAPLPT---PAAPQQIISPVN--PAWQHHHYFPPSSTTP
N.fischeri     214 PAPNTFQ--NPGS---FPPTSAASLPS----PGHQQMISPVT--PAWQHHHYFPPSSSTP
P.chrysogenum  168 PTTSSFQPSTPASAPQMPPVQVQTQQPHSHSHSSSALISPVT--PAWQHHHYFPPSTTAP
C.immitis      221 ---------RPVT---FPPVAFAVLPS---QPTHPQVLPLGS--PAWQHHHYFPPSNTAT
A.capsulatus   232 SHPPSFSTSIPLA-----------APP-----AQQHHHHSMI--STWQHHHYFPPSNTAP
U.reesii       226 ---------RPTN---FPPVTFAVLPS---QPTHPQMVALGS--PAWQHHHYFPPSNTAT
P.marneffei    131 AYPRSHSTSAPVT-------PSTLVPP------PPAMLSPVNH-PGWQHHHYFPLSTTTS
B.fuckeliana   211 ---------LPQQ---FP-------PP---MMPVSVTLTPSSGANPWQHHHYISPSSAAS
N.tetrasperma  189 ---------LPQT---FT-------PA----VPVPVTLAPVTGANPWQHHHYIAPSSTAS
N.crassa       189 ---------LPQT---FT-------PA----VPVPVTLAPVTGANPWQHHHYIAPSSTAS
M.oryzae       200 ---------LPQA---F--------PP----MPVNVSMGPQSGANPWQHHHYISPSAAAS
C.globosum     223 ---------LPQS---FP-------PP----MSMSMTLAPTAG-NPWQHHHYIAPSASAS
F.oxysporum    170 ---------LPQA---YP-------PP------VSMPAPAPSGANPWQHHHYLNPTGAAA

A.nidulans     243 YQQNHDRYICRTCHKAFSRPSSLRIHSHSHTGEKPFRCTHAGCGKAFSVRSNMKRHERGC
A.oryzae       249 YQQNHDRYICRTCHKAFSRPSSLRIHSHSHTGEKPFRCTHAGCGKAFSVRSNMKRHERGC
A.niger        255 YQQNHDRYICRTCHKAFSRPSSLRIHSHSHTGEKPFRCTHAGCGKAFSVRSNMKRHERGC
A.kawachii     255 YQQNHDRYICRTCHKAFSRPSSLRIHSHSHTGEKPFRCTHAGCGKAFSVRSNMKRHERGC
N.fischeri     263 YQQNHDRYICRTCHKAFSRPSSLRIHSHSHTGEKPFRCTHAGCGKAFSVRSNMKRHERGC
P.chrysogenum  226 YQQNHDRYICRTCHKAFSRPSSLRIHSHSHTGEKPFRCTHAGCGKAFSVRSNMKRHERGC
C.immitis      264 YPLNHDRYICRICHKAFSRPSSLRIHSHSHTGEKPFRCPHAGCGKAFSVRSNMKRHERGC
A.capsulatus   274 YPQNHDRYICRICHKAFSRPSSLRIHSHSHTGEKPFKCPHVNCGKSFSVRSNMKRHERGC
U.reesii       269 YPLNHDRYICRICHKAFSRPSSLRIHSHSHTGEKPFRCPHAGCGKAFSVR-NQPRSQRSL
P.marneffei    177 YPQNHERYVCRTCHKAFSRPSSLRIHSHSHTGEKPFRCTHAGCGKAFSVRSNMKRHERGC
B.fuckeliana   249 FPQSQDRYICQTCNKAFSRPSSLRIHSHSHTGEKPFKCPHQNCGKAFSVRSNMKRHERGC
N.tetrasperma  226 FPQSQDRYICQTCNKAFSRPSSLRIHSHSHTGEKPFKCPHAGCGKAFSVRSNMKRHERGC
N.crassa       226 FPQSQDRYICQTCNKAFSRPSSLRIHSHSHTGEKPFKCPHAGCGKAFSVRSNMKRHERGC
M.oryzae       236 FPQSQDRYICQTCNKAFSRPSSLRIHSHSHTGEKPFKCPHAGCGKAFSVRSNMKRHERGC
C.globosum     259 FPQSQDRYICPTCSKAFSRPSSLRIHSHSHTGEKPFKCPFPGCGKAFSVRSNMKRHERGC
F.oxysporum    205 FPQSQDRYICPTCNKAFSRPSSLRIHSHSHTGEKPFKCPHAGCGKAFSVRSNMKRHERGC

A.nidulans     303 HTGRAVAM---------------------------------V------------------
A.oryzae       309 HTGRPVAT---------------------------------AMV----------------
A.niger        315 HSGRPVAT---------------------------------AMV----------------
A.kawachii     315 HSGRPVAT---------------------------------AMV----------------
N.fischeri     323 HTGRPVAT---------------------------------AMVQ---------------
P.chrysogenum  286 HSGRPAPAPAAT-----------------------------ALVV---------------
C.immitis      324 HPGRSAPPS--------------------------------ALVN---------------
A.capsulatus   334 HTGRPTQA---------------------------------ALVN---------------
U.reesii       328 IEKRKGYAIGFDEWVLTMITPTIRSTNEQIYTTASCKIANVAVININRRIAELRKSFRNR
P.marneffei    237 HSGRPMTA---------------------------------TVV----------------
B.fuckeliana   309 HSFESASMV---------------------------------------------------
N.tetrasperma  286 HSFESSNGRSSGNSNNGASA----------------------------------------
N.crassa       286 HSFESSNGRSSGNSNNSASA----------------------------------------
M.oryzae       296 HNYDSSSSNGTAMH----------------------------------------------
C.globosum     319 HNYDSSSTTSSTGTMNSNTGGSRP------------------------------------
F.oxysporum    265 HSFEFNGSVIRG------------------------------------------------

A.nidulans         ------------------------------------
A.oryzae           ------------------------------------
A.niger            ------------------------------------
A.kawachii         ------------------------------------
N.fischeri         ------------------------------------
P.chrysogenum      ------------------------------------
C.immitis          ------------------------------------
A.capsulatus       ------------------------------------
U.reesii       388 RSNGTLSPTKRRVKLAFSLDCQSTSSSRLALLPQSL
P.marneffei        ------------------------------------
B.fuckeliana       ------------------------------------
N.tetrasperma      ------------------------------------
N.crassa           ------------------------------------
M.oryzae           ------------------------------------
C.globosum         ------------------------------------
F.oxysporum        ------------------------------------
