# Supplementary figures and images for: The Putative C2H2 Transcription Factor MtfA Is a Novel Regulator of Secondary Metabolism and Morphogenesis in Aspergillus nidulans
Source: PLoS One. 2013 Sep 16;8(9):e74122. doi: 10.1371/journal.pone.0074122 (PMC3774644; doi:10.1371/journal.pone.0074122)

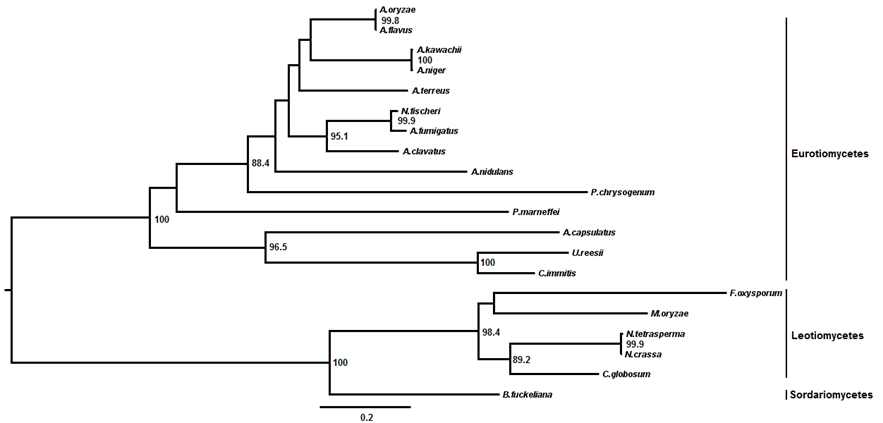

Supplement: Figure S2 — Maximum-Likelihood (ML) phylogenetic tree inferred from ortholog sequences of MtfA ( A.nidulans ) across genomes from several fungal species. Protein alignment was done with MUSCLE; aLRT (approximate Likelihood Ratio Test) branch support values were calculated with PhyML v3.0 and the tree was plotted using FigTree v1.4.0. Only alRT branch support values >80% are indicated. The protein sequences used are as follows: Aspergillus oryzae (A.oryzae), Aspergillus flavus (A.flavus), Aspergillus kawachii (A.kawachii), Aspergillus niger (A.niger), Aspergillus terreus (A.terreus), Neosartorya fischeri (N.fischeri), Aspergillus fumigatus (A.fumigatus), Aspergillus clavatus (A.clavatus), Aspergillus nidulans (A.nidulans), Penicillium chrysogenum (P.chrysogenum), Penicillium marneffei (P.marneffei), Ajellomyces capsulatus (A.capsulatus), Uncinocarpus reesii (U.reesii), Coccidioides immitis (C.immitis), Fusarium oxysporum (F.oxysporum), Magnaporthe oryzae (M.oryzae), Neurospora tetrasperma (N.tetrasperma), Neurospora crassa (N.crassa), Chaetomium globosum (C.globosum) and Botryotinia fuckeliana (B.fuckeliana). NCBI (National center for Biotechnology Information) accession numbers for all sequences utilized in these analyses are shown in Table S1 in the supplemental material. (TIF) [file pone.0074122.s002.tif]

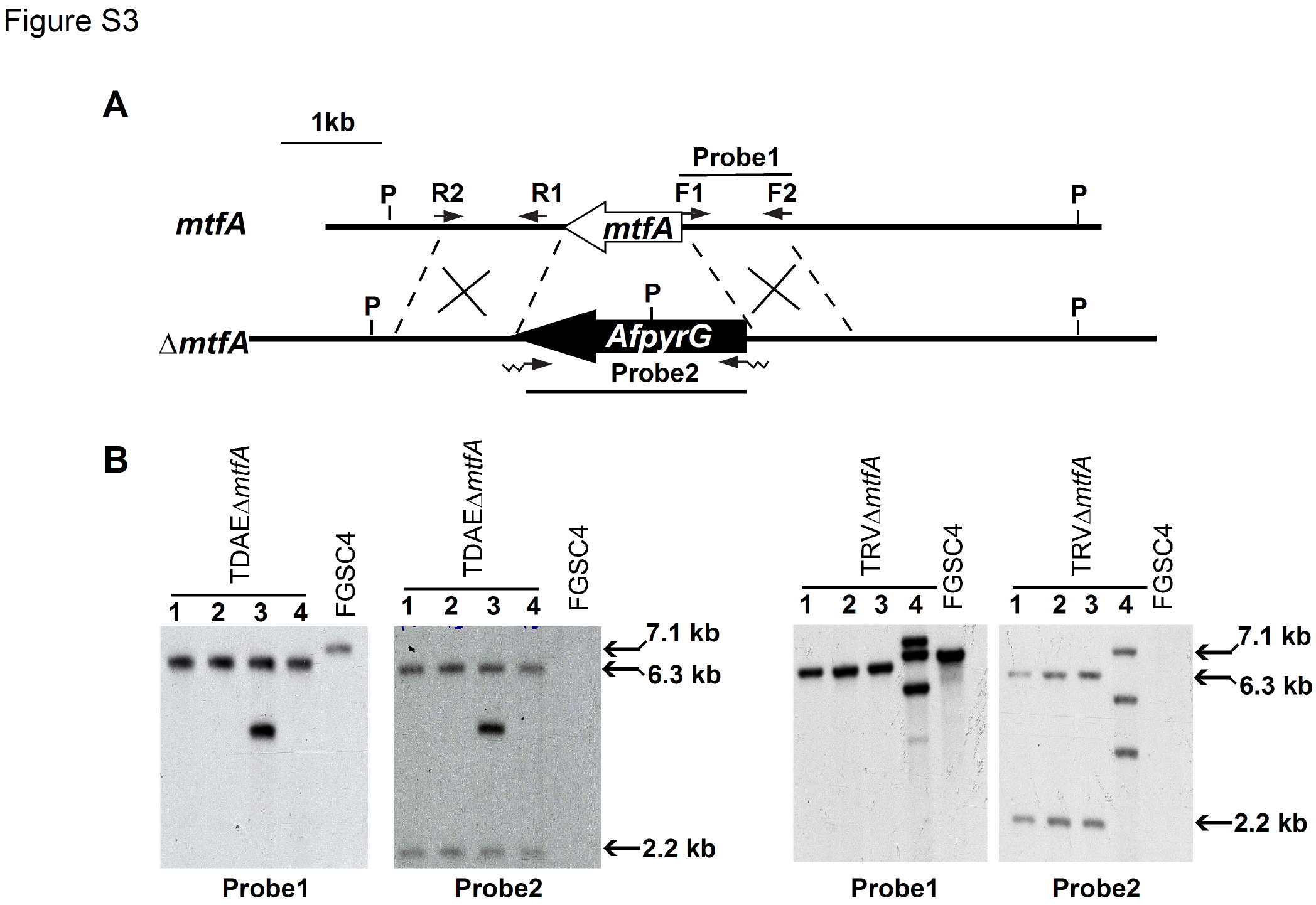

Supplement: Figure S3 — Targeted mtfA deletion. A) Diagram showing PstI sites (P) in the wild-type mtfA locus, and the same locus after gene replacement of mtfA by the A. fumigatus pyrG gene (AfpyrG), used as selection marker for fungal transformation. Recombination events between the the flanking regions are indicated with crosses (X). Primers used for the construction of the deletion cassette are indicated by small arrows as described by FGSC. Fragments used as probe templates for Southern blot analyses are also shown. B) Southern blot analyses. The ΔmtfA deletion construct was transformed in RDAE206 and RJMP 1.49 strains (Table 1). PstI digested genomic DNA of FGSC4 wild type (WT) and transformants, TDAEΔmtfA (ΔveA, ΔmtfA) and TRVΔmtfA (veA+ ΔmtfA), was hybridized with probe P1, containing 5′ flanking sequence of mtfA, and probe P2, containing AfpyrG coding fragment. TDAEΔmtfA transformants #1, 2 and 4 present the correct band pattern. TRVΔmtfA transformants #1, 2 and 3 present the correct band pattern. (TIF) [file pone.0074122.s003.tif]

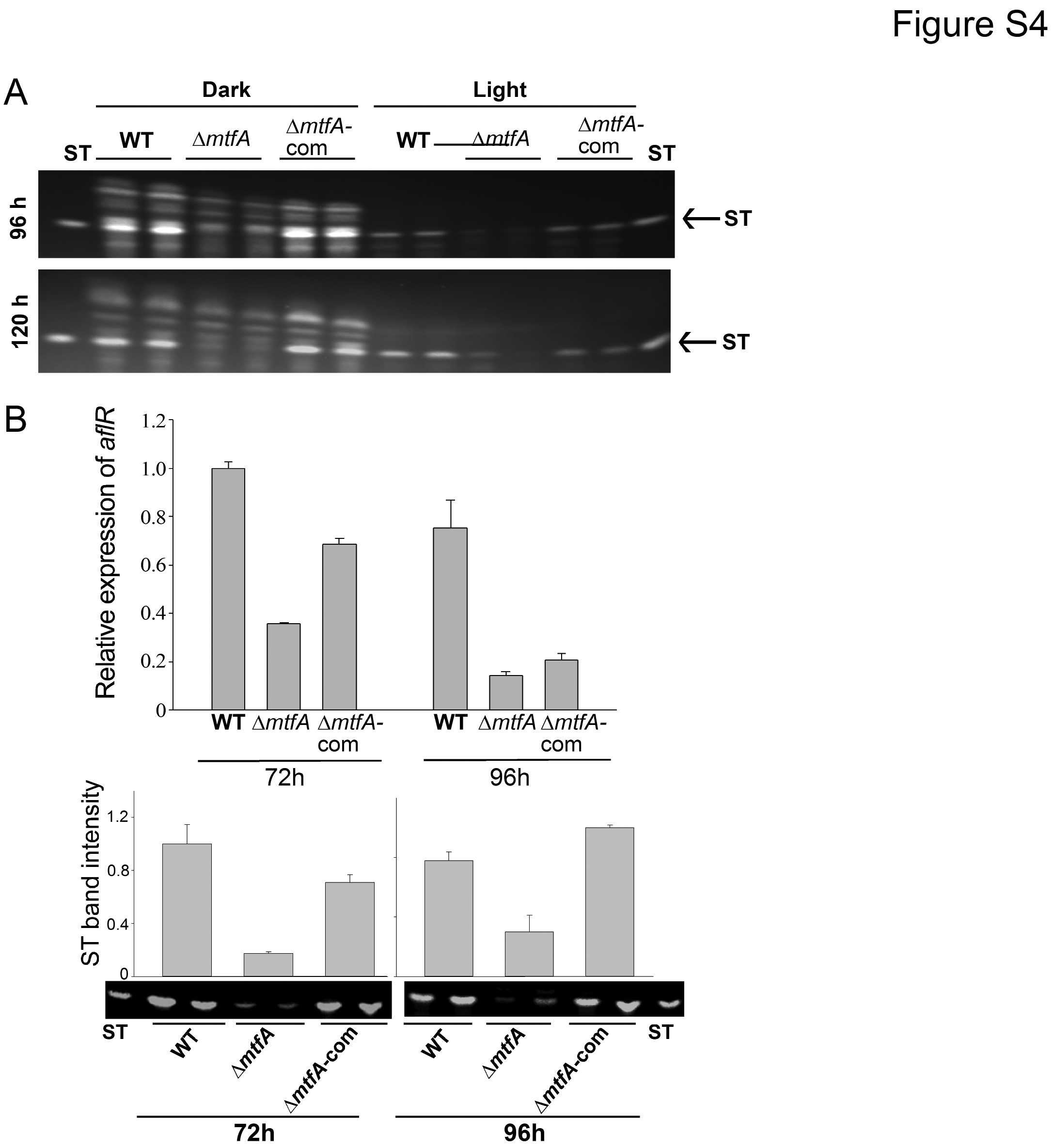

Supplement: Figure S4 — Effects of mtfA deletion on ST production and aflR expression at late time points. A) TLC analysis showing ST production in GMM cultures. Wild type (WT) veA+ control (TRV50.2), ΔmtfA (TRVpΔmtfA) and ΔmtfA-com complementation strain (TRVΔmtfA-com) were spread-inoculated with 5 mL of top agar containing 106 conidia mL−1 and incubated at 37°C in the dark or in the light for 96 h and 120 h. ST was extracted and analyzed by TLC. B) Effect of the mftA deletion on aflR expression. Wild type (WT) veA+ control (TRV50.2), ΔmtfA (TRVpΔmftA) and ΔmtfA-com complementation strain (TRVΔmtfA-com) were inoculated in liquid GMM. Mycelia were collected 72 h and 96 h after inoculation. Cultures were grown in a shaker incubator at 37°C at 250 rpm. Expression of aflR was analyzed by qRT-PCR. A TLC showing accumulation of ST in these cultures and corresponding densitometry is also shown. (TIF) [file pone.0074122.s004.tif]

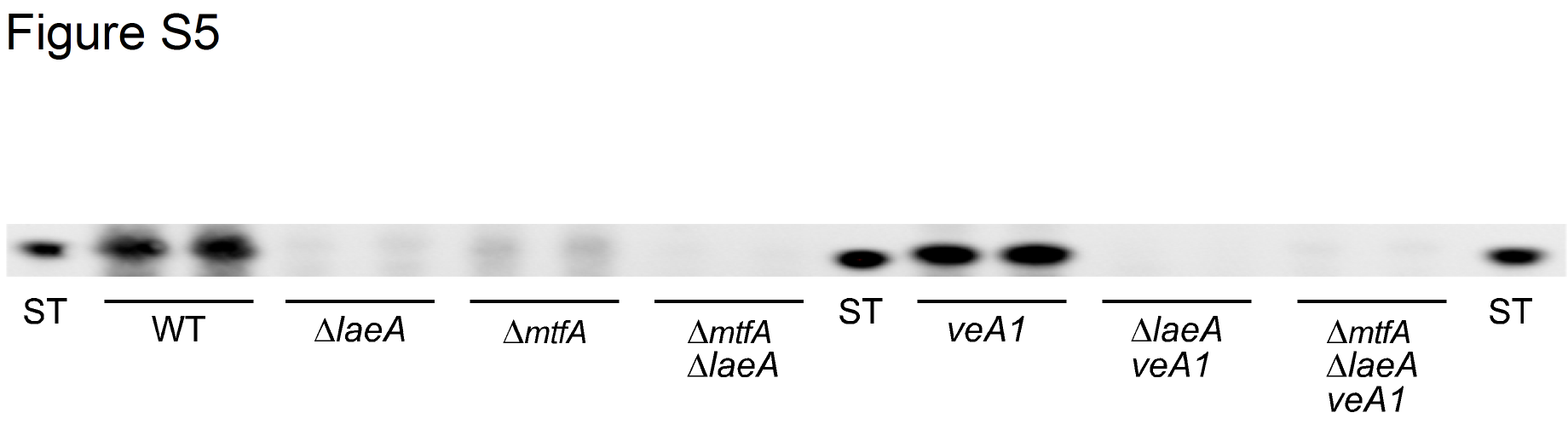

Supplement: Figure S5 — Deletion of mtfA does not rescue mycotoxin production in Δ laeA strains. TLC analysis of ST produced by the wild type (WT) veA+ control (TRV50.2), ΔlaeA veA+ (RJW41.A), ΔmtfA veA+ (TRVpΔmtfA) and ΔmtfA ΔlaeA veA+ strains (RSD11.2), veA1 (RDIT2.3), ΔmtfA veA1 (RJW46.4), ΔmtfA ΔlaeA veA1 (RSD10.1) grown on GMM at 37°C for 5 days. (TIF) [file pone.0074122.s005.tif]

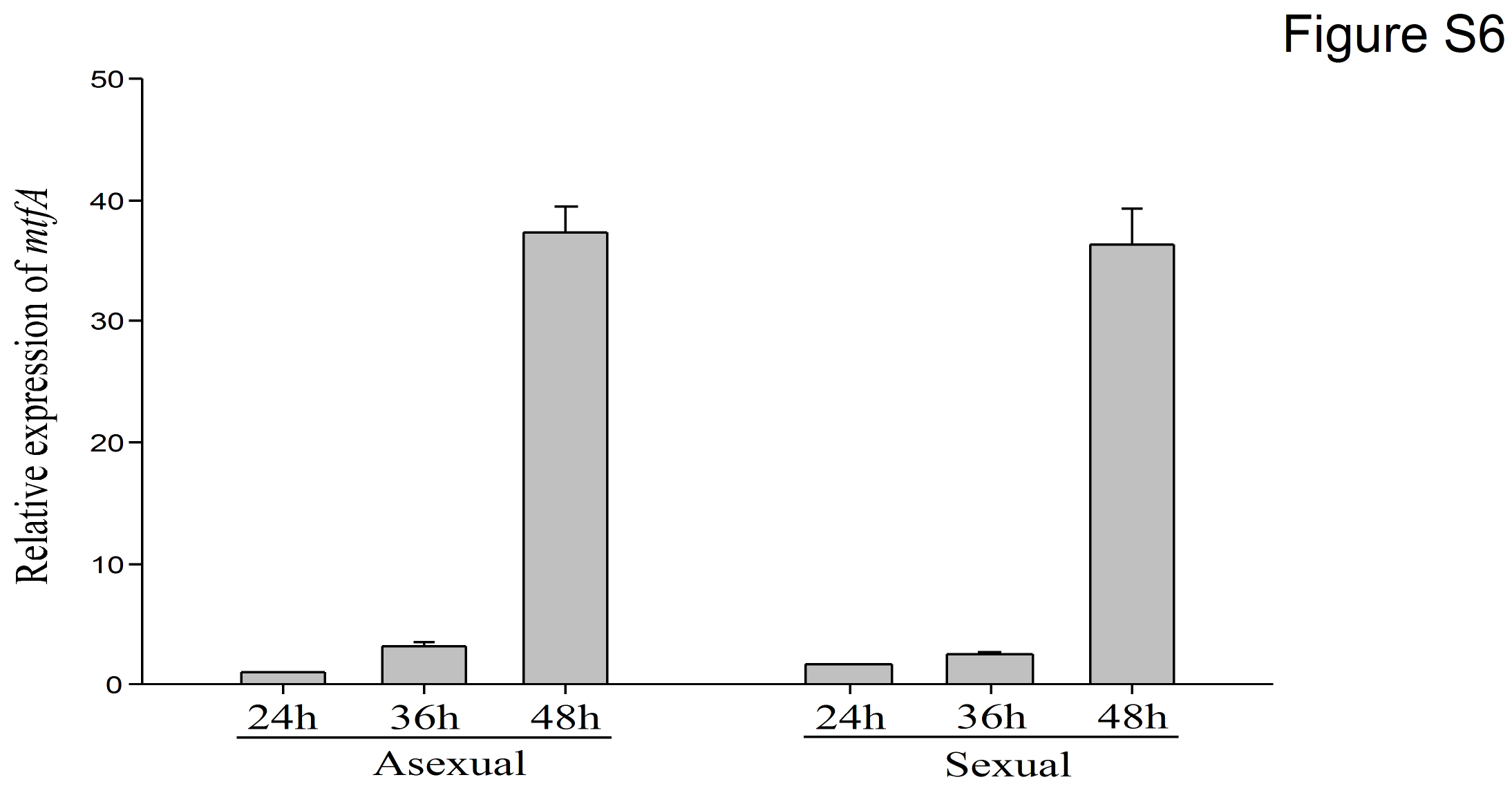

Supplement: Figure S6 — Expression of mtfA in the wild-type strain. qRT-PCR analysis showing mtfA expression in the wild-type strain (TRV50.2) at the times indicated under conditions promoting asexual (light) or sexual development (dark). The strains were top-agar inoculated on GMM and incubated at 37°C. (TIF) [file pone.0074122.s006.tif]

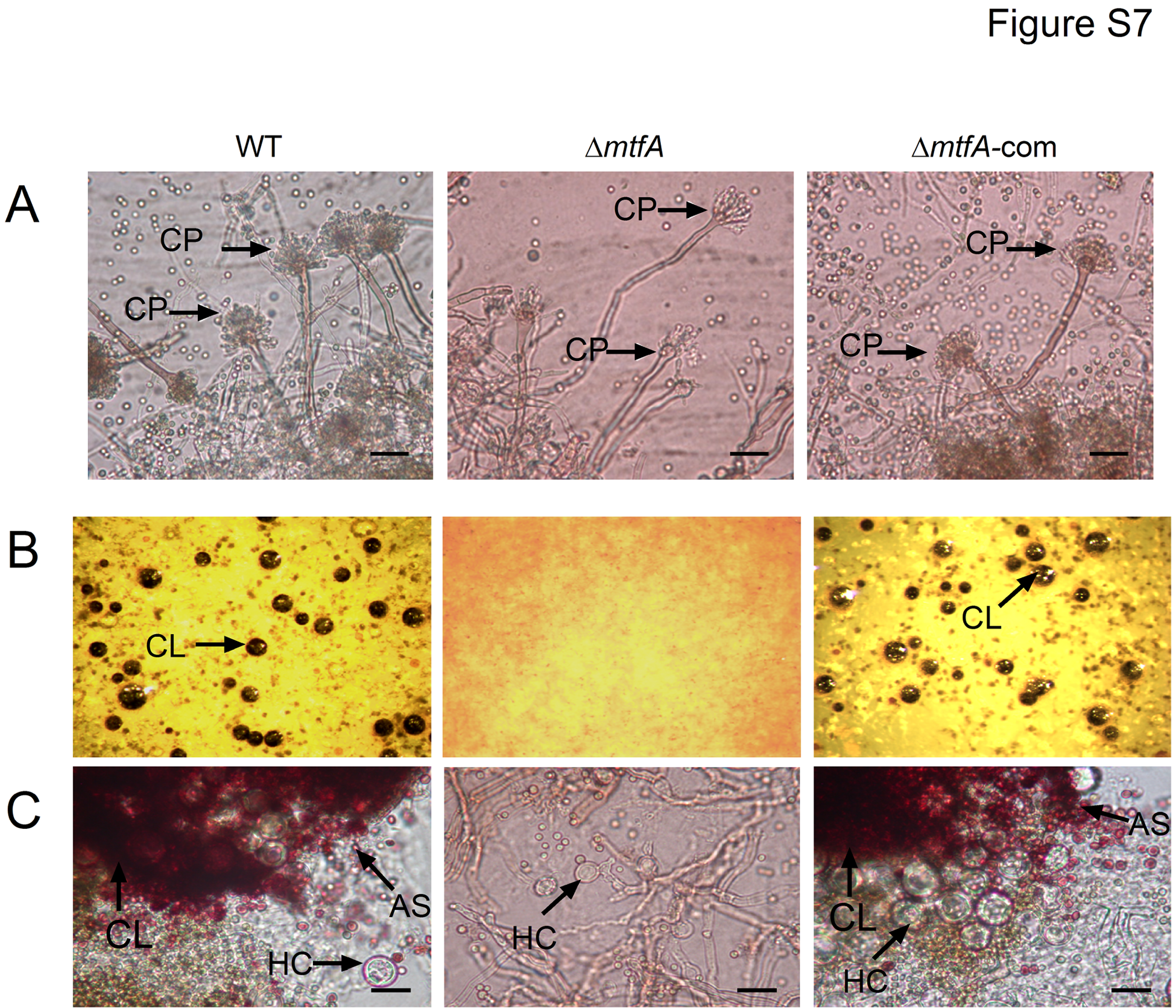

Supplement: Figure S7 — Micrographs of asexual and sexual structures. A) Conidiophores forming in wild type (WT) veA+ (TRV50.2), ΔmtfA (TRVpΔmtfA) and ΔmtfA-com complementation (TRVΔmtfA-com) strains in top agar-inoculated solid GMM cultures incubated for 5 days in the light at 37°C. Bar represent 20 micrometers. CP, conidiophores. B) Micrographs showing the presence of cleistothecia (CL) in wild type (WT) veA+ (TRV50.2), and ΔmtfA-com complementation (TRVΔmtfA-com) cultures growing in the dark for 5 days. Magnification 50×. C) Micrographs showing details of sexual structures. Bar represents 15 micrometers. CL, portion of an open cleistothecium; AS, ascospores; HC, Hülle cells. (TIF) [file pone.0074122.s007.tif]
