# Supplementary material for: The Putative C2H2 Transcription Factor MtfA Is a Novel Regulator of Secondary Metabolism and Morphogenesis in Aspergillus nidulans
Source: PLoS One. 2013 Sep 16;8(9):e74122. doi: 10.1371/journal.pone.0074122 (PMC3774644; doi:10.1371/journal.pone.0074122)
Supplement: Table S1 — Amino acid sequence comparison of Aspergillus nidulans MtfA in with putative orthologs in other fungal species. The comparisons were done using the BLASTp tool provided by NCBI (National Center for Biotechnology Information) and EMBOSS Needle - Pairwise Sequence Alignment tool provided by EMBL-EBI (European Bioinformatics Institute). (DOCX) [file pone.0074122.s008.docx]

**Table S1**: Amino acid sequence comparison of *Aspergillus nidulans* MtfA in with putative orthologs in other fungal species. The comparisons were done using the BLASTp tool provided by NCBI (National Center for Biotechnology Information) and EMBOSS Needle - Pairwise Sequence Alignment tool provided by EMBL-EBI (European Bioinformatics Institute).

| **Name of the species, with the strain information** | **NCBI** | |  | **EMBOSS Needle - Pairwise Sequence Alignment**  **(global alignment)** | | |
| --- | --- | --- | --- | --- | --- | --- |
|  | **Accession number** | **E-value (BLASTp)** |  | **Length** | **%Identity** | **%Similarity** |
| *Aspergillus oryzae* [RIB40] | XP_001823905.1 | 0 |  | 332 | 64.2 | 70.8 |
| *Aspergillus clavatus* [NRRL 1] | XP_001270264.1 | 2E-111 |  | 347 | 65.1 | 71.2 |
| *Aspergillus niger* [CBS 513.88] | XP_001395874.1 | 5E-106 |  | 336 | 62.8 | 71.1 |
| *Aspergillus kawachii* [IFO 4308] | GAA87693.1 | 6E-106 |  | 336 | 62.8 | 70.8 |
| *Aspergillus fumigatus* [Af293] | XP_747808.1 | 2E-100 |  | 342 | 62 | 71.3 |
| *Neosartorya fischeri* [NRRL 181] | XP_001257459.1 | 5E-94 |  | 353 | 60.9 | 68.8 |
| *Aspergillus flavus* [NRRL3357] | XP_002380969.1 | 9E-94 |  | 332 | 64.2 | 70.8 |
| *Aspergillus terreus* [NIH2624] | XP_001209872.1 | 6E-93 |  | 344 | 62.5 | 68.9 |
| *Penicillium chrysogenum* [Wisconsin 54-1255] | XP_002566301.1 | 3E-74 |  | 351 | 49.3 | 58.7 |
| *Coccidioides immitis* [RS] | XP_001239027.1 | 1E-64 |  | 355 | 44.5 | 54.6 |
| *Ajellomyces capsulatus* [H88] | EGC49893.1 | 9E-64 |  | 364 | 45.9 | 58.0 |
| *Uncinocarpus reesii* [1704] | XP_002585289.1 | 6E-54 |  | 440 | 34.1 | 42.0 |
| *Penicillium marneffei* [ATCC 18224] | XP_002148846.1 | 1E-52 |  | 342 | 38 | 43.9 |
| *Botryotinia fuckeliana* | CCD44702.1 | 6E-47 |  | 347 | 40.3 | 51.9 |
| *Neurospora tetrasperma* [FGSC 2508] | EGO52630.1 | 2E-44 |  | 347 | 39.8 | 50.1 |
| *Neurospora crassa* [OR74A] | XP_964590.1 | 2E-44 |  | 343 | 39.1 | 50.1 |
| *Magnaporthe oryzae* [70-15] | XP_003720663 | 4E-50 |  | 335 | 38.5 | 50.4 |
| *Chaetomium globosum* [CBS 148.51] | XP_001222401.1 | 6E-39 |  | 382 | 34.0 | 45.8 |
| *Fusarium oxysporum* [Fo5176] | EGU84033.1 | 3E-38 |  | 350 | 34.9 | 43.4 |
