# Supplementary material for: The Putative C2H2 Transcription Factor MtfA Is a Novel Regulator of Secondary Metabolism and Morphogenesis in Aspergillus nidulans
Source: PLoS One. 2013 Sep 16;8(9):e74122. doi: 10.1371/journal.pone.0074122 (PMC3774644; doi:10.1371/journal.pone.0074122)
Supplement: Table S2 — Comparison of MtfA with other A. nidulans C2H2 transcription factors. (DOC) [file pone.0074122.s009.doc]

**Table S2. Comparison of MtfA with other *A. nidulans* C_2_H_2_ transcription factors**

**Transcription Factor Accession No. (NCBI)%identitylength%identity of the DNA binding domain**FlbCACP2886725.339929.0BrlAXP_65857721.445719.2SteAO7425211.374227.9PacCCAA873906.18309.8SltAXP_66052311.772021.7CrzAXP_66333014.374625.8CreAAAR028586.460725.0Note: The pairwise sequence alignment was carried out with EMBOSS Needle, provided by EMBL-EBI.
